# Ferulic acid: a liver protector

**DOI:** 10.3389/fphar.2026.1860327

**Published:** 2026-07-29

**Authors:** Yuqiu Zhang, Xinghe Wang, Xinyue Sun, Jianan Dai

**Affiliations:** 1 Suining County Traditional Chinese Medicine Hospital, Xuzhou, Jiangsu, China; 2 College of Animal Science and Veterinary Medicine, Shenyang Agricultural University, Shenyang, Liaoning, China; 3 Heilongjiang University of Chinese Medicine, Harbin, Heilongjiang, China

**Keywords:** antioxidant, ferulic acid, liver damage, prevention, treatment

## Abstract

Ferulic acid (FA) is a phenolic acid found in plants, such as wheat, corn, beet, etc. It is an important active ingredient in many herbal medicines. FA has been reported to have a variety of biological activities, especially in the resistance to oxidative stress, inflammation, vascular endothelial damage, fibrosis, apoptosis, and platelet aggregation. Because FA has advantages of high activities, low cost, non-toxic, good safety and stable structure. So it is widely used in food additives, medicine, healthcare products, and cosmetics. Numerous studies have shown the protective effects of FA and its derivatives on the liver. This paper reviews the recent research progress of FA and its derivatives in preventing and curing liver damages induced by various different factors, including the effects of FA and its derivatives on physicochemical factors induced liver injury, liver tumors, alcoholic liver disease, non-alcoholic fatty liver disease, liver metabolic disorder of diabetics, and hepatotoxicity of aflatoxin B1. The aim of this review is to provide reference for the research and application of FA and its derivatives in the prevention and treatment of liver diseases.

## 1 Introduction

**FIGURE 1:**
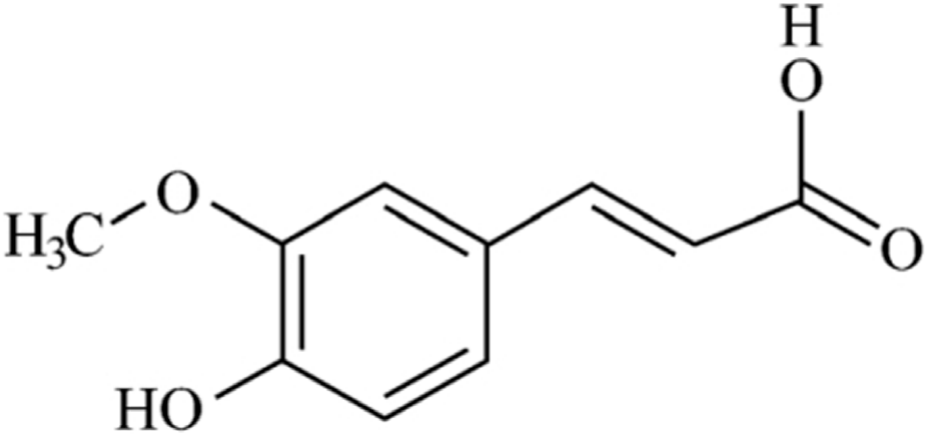
The chemical structure of ferulic acid (adapted from Kiokias and Oreopoulou, 2021, licensed under CC BY 4.0).

Ferulic acid (FA) is a cinnamic acid derivative with the formula C_10_H_10_O_4_, relative molecular mass of 194.19 (Kiokias and Oreopoulou, 2021). As shown in Figure 1, the double bond, hydroxyl, and methoxy in the chemical structure of FA are the main active groups. The chemical structure of FA has both in cis and in trans. In practical, trans-FA is used more often, and it is white or slightly yellow square crystallization or fiber crystallization. Because FA has high activities, low toxicity and low cost, so it is widely used in food additives, medicine, health products, cosmetics, etc.

As the basic component of plant cell wall, FA combines with polysaccharides and proteins to form the skeleton of plant cell wall. FA is widely existing in edible plants, such as tomatoes, sweet corn, rice bran, etc. It is the main combined phenolic acid in sweet corn, accounting for 78% of the total phenolic acid. FA is one of the effective ingredients of traditional Chinese medicines. The content of FA in some traditional Chinese medicines is very high, especially Ferula officinalis, Angelica sinensis, Ligusticum chuanxiong, and Semen ziziphi spinosae. FA has significant antioxidant and free radical scavenging effects. In addition, FA also has pharmacological effects and biological activities such as anti-mutagenic, anti-inflammatory, protect vascular endothelial, immune enhancement, anti-fibrosis, anti-apoptosis, and reducing platelet aggregation (Rukkumani et al., 2004; Panneerselvam et al., 2013; Katayama et al., 2017; Ezhuthupurakkal et al., 2018; Kiokias and Oreopoulou, 2021). FA can inhibit the phosphatidylinositol-3-kinase (PI3K)/protein kinase B (AKT) pathway, reduce reactive oxygen species (ROS) and malondialdehyde (MDA) production, promote the formation of antioxidants, and inhibit the activity of aldose reductase (He et al., 2019; Akinyelu et al., 2025). The anti-inflammatory effects of FA are related to the levels of peroxisome proliferator-activated receptor γ (PPARγ), cell adhesion molecule (CAM), nuclear factor κ B (NF-κB), and p38 mitogen-activated protein kinase (p38 MAPK) (Yin et al., 2019; Shi et al., 2023). FA not only protects the vascular endothelium through extracellular regulatory protein kinase (ERK 1/2) and vasoactive substances (NO/ET-1) signaling pathways, but also acts as an antifibrotic effect through transforming growth factor-β (TGF-β)/*drosophila* mothers against decapentaplegic protein (Smad) and matrix metalloproteinases (MMPs)/tissue metalloproteinase inhibitors (TIMPs) systems (Mu et al., 2018; Li et al., 2021). Moreover, FA also has anti-apoptotic and antiplatelet effects. It is thought to involve in preventing cancer, colds, influenza, skin aging, and muscle atrophy (Rukkumani et al., 2004; Kiokias and Oreopoulou, 2021).

In recent years, the health protective effect of FA has received much attention (Kiokias and Oreopoulou, 2021; Shi et al., 2023), but its protective effect on liver is still in the early stage of research. To provide a scientific basis for the development and application of FA in the field of liver protection, this paper will review the current status of the liver protective effect of FA.

## 2 Hepoprotective effect of ferulic acid and its derivatives

### 2.1 Protective effect of ferulic acid and its derivative on liver injury induced by physicochemical factors

#### 2.1.1 Ferulic acid, ferulic acid derivatives, and nano-formulated ferulic acid protect liver from classic hepatoxicants (CCl_4_, acetaminophen, and mycotoxin)

##### 2.1.1.1 Ferulic acid and nano-formulated ferulic acid alleviate carbon tetrachloride-induced liver injury

Some scholars have studied the protective effect of FA on carbon tetracloride (CCl_4_) induced acute liver injury. Intraperitoneal injection of ferulic acid (20, 40, 80 mg/kg) 1 h before and 2 h after CCl_4_ (20 mL/kg) injection, respectively (Kim et al., 2011). After CCl_4_ injection, serum transaminase activity, tumour necrosis factor (TNF) level, and liver MDA level were significantly increased. The prototype glutathione (GSH) concentration in serum was reduced. FA administration attenuated these changes induced by CCl_4_. The mRNA expression levels of inducible nitric oxide synthase (iNOS) and cyclooxygenase-2 (COX-2) protein expression in CCl_4_ treated liver were significantly increased. While FA treatment reduced CCl_4_ induced high expression of iNOS and COX-2. In addition, CCl_4_ significantly increased c-Jun terminal kinase (JNK) phosphorylation and p38 MAPK level, as well as the nuclear translocation of activated c-Jun. FA administration reduced the high levels of JNK phosphorylation, p38 MAPK, and inhibited the nuclear translocation of activated c-Jun. It was suggested that FA could improve the antioxidant level of hepatocytes through iNOS and COX-2, and inhibit hepatic inflammation through JNK and p38 MAPK signaling pathways, thus exerting hepatoprotective effect in CCl_4_-induced liver injury. Another research showed that acute CCl_4_ stimulation induced the high transcription and expression of TLR4, TLR2, and TLR9. FA significantly inhibited the high expression of TLR4 induced by CCl_4_ (Kim et al., 2011). Early studies using rats showed that FA alleviated CCl_4_-induced liver damage via reducing the accumulation of cholesterol, triglycerides (TG), free fatty acids (FFA), thiobarbituric acid-reactive substances (TBARS), hydroperoxides (HP), NO, protein carbonyl content (PCO) in liver, and elevating phospholipids (PL), SOD, CAT, GPx, and GSH levels in liver (Srinivasan et al., 2005; Marimuthu et al., 2013). These parameters changes proved that FA could alleviate CCl_4_-induced liver injury through balancing lipid metabolism and elevating the antioxidant capacity of liver. A recent study improved the hepatoprotective effect of FA by loading FA into polyglycerol polymeric nanoparticles (Mahmoud et al., 2024). Methyl ferulic acid (MFA) is a derivative of FA, which is a monomer can be extracted and purified from the rhizome of Securidaca inappendiculata Hasskarl. Oral administration of MFA (25, 50, and 100 mg/kg) protected rat liver from CCl_4_-induced damage via down-regulating ALT, AST, ROS, MDA, NADPH oxidase (NOX) trans-membrane subunit NOX4, p22^phox^, TBARS, TNF-α, interleukin-1β (IL-1β), Bcl-2-associated X protein (Bax)/B-cell lymphoma (Bcl-2), cleaved caspase3/caspase3, phosphorylated J-Jun N-terminal kinase (p-JNK)/JNK, and p-p38/p38; and up-regulating SOD, CAT, GSH-Px, and total anti-oxidant capacity (TAC) (Yang et al., 2018). These findings suggested that FA, MFA, and nano-formulated ferulic acid alleviate CCl_4_-induced acute liver injury by reducing oxidative damage, enhancing anti-oxidant capacity, inhibiting apoptosis, and regulating inflammatory signaling pathways (Table 1).

**TABLE 1:** Protective effect of ferulic acid and its derivative on liver injury induced by physicochemical factors.

FA or FA derivative or nano-formulated FA	Physicochemical factors	Prevention or treatment	FA or its derivative regulated signaling pathway	Apparent change	Animal or cell	References
FA	CCl_4_	Prevention	GSH/GSSG ↑, MDA ↓ TLR2, TLR4, TLR9, TNF-α, iNOS, COX-2 ↓ NF-κB nuclear translocation ↓ MAPK pathway (JNK1, JNK2, ERK, p38, AP-1) ↓	Histopathological changes ↓ Oxidative damage ↓ Inflammation ↓	Mouse	Kim et al. (2011)
FA	CCl_4_	Prevention	cholesterol, TG, FFA ↓ phospholipids (PL) ↑	Histopathological changes ↓	Rat	Marimuthu et al. (2013)
FA	CCl_4_	Prevention	SOD, GSH ↑ MDA, ALT, AST, ALP, TP ↓	Histopathological changes ↓	Mouse	Mahmoud et al. (2024)
Polyglycerol polymeric nanoparticles loaded with FA	CCl_4_	Prevention	SOD, GSH ↑ MDA, ALT, AST, ALP, TP ↓	Histopathological changes ↓	Mouse	Mahmoud et al. (2024)
FA	CCl_4_	Prevention	ALT, AST, ALP, GGT, TBARS, HP, NO, PCO ↓ SOD, CAT, GPx, GSH ↑	Antioxidant capacity↑ Lipid peroxidation ↓	Rat	Srinivasan et al. (2005)
MFA	CCl_4_	Prevention	ALT, AST ↓ SOD, CAT, GSH-Px ↑ ROS, MDA, NOX4, p22^phox^, TBARS ↓ TNF-α, IL-1β ↓ Bax/Bcl-2, cleaved caspase3/caspase3, p-JNK/JNK, p-p38/p38 ↓	Histopathological changes ↓ TAC ↑ Inflammation score ↓ Cell apoptosis ↓	Rat	Yang et al. (2018)
FA	Acetaminophen	Prevention	ALT, AST, MDA, Cleaved caspase3, Bax, P62 ↓ SOD, Bcl-2, ATG5, LC3-II/LC3-I, p-AMPK ↑	Liver necrosis ↓ Apoptosis ↓ Oxidative damage ↓ Histopathological score↓ Autophagy ↑	Mouse	Wu et al. (2022)
FA	Acetaminophen	Prevention	Hnf4α/Hprt1, Foxa2/Hprt1, ALB/TP ↑ Glycogen, SOD ↑ MDA, Cleaved caspase3, Bax, P62↓ Bcl-2, ATG5, LC3-II/LC3-I ↑	Cell viability ↑ Cell apoptosis ↓ Autophagy ↑	Mouse primary hepatocyte	Wu et al. (2022)
FA	Acetaminophen	Prevention	ALT, AST, Caspase3, CYP2E1 ↓ SOD, CAT, GSH ↑ TLR4, TNF-α, IL-1β, p-IRAK1, p-IκB, p-P38 ↓	Histopathological changes ↓ Inflammation ↓ Apoptosis ↓	Mouse	Yuan et al. (2016)
SF	Acetaminophen	Prevention	ALT, MDA, Glycogen and glutathion ↓ GST ↑	The membrane fluidity of liver microsome and the mitochondria ↓	Mouse	Wang and Peng (1994)
FA	Aflatoxin B1	Prevention	ALT, AST, GGT, ALP, TBA, ROS, MDA ↓ CYP1A2, CYP2A6, CYP3A4, CYP2E1 ↓ Nrf2, SOD, GST, bcl-2↑ p53, bax, cyt-c, caspase-9, caspase-3 ↓ The amino acids of GLN360, ASP364, PRO431, and ALA445 in CYP2A6 protein can be bound by both AFB1 and FA	AFBO formation ↓ Morphological changes ↓ Apoptosis ↓	Rat liver	Wang Z. et al. (2021)
FA	Aflatoxin B1	Prevention	GST, Claudin-1, ZO-1 ↑ ROCK1, ROCK2, CYP2A6, CYP3A4 ↓ Binding affinity of FA to CYP2A6 is very similar to the binding affinity of AFB1 to CYP2A6	Morphological changes ↓ Apoptosis ↓ Tight junction ↑	Rat small intestine	Wang X. et al. (2022)
FA	Aflatoxin B1 + Ochratoxin A	Prevention	4-HNE, Polyubiquitinated Nrf2 ↓ SOD ↑ ROS ↓ to preserve thiol groups	Oxidative stress ↓	Caco-2 cells	Rădulescu et al. (2025)
FA in brown rice	Aflatoxin B1	Prevention	Liver index, Spleen index, Kidney index ↓ AST, ALT, MDA, Caspase3, Bax/Bcl-2 ↓ Nrf2, HO-1, SOD, T-AOC ↑	Histopathological changes ↓ Antioxidant capacity ↑ Apoptosis ↓ Body weight ↑	Mouse	Wang et al. (2025)
FA in brown rice	Aflatoxin B1	Prevention	AST, ALT, LDH, MDA, ROS ↓ SOD, T-AOC, MMP ↑	Antioxidant capacity ↑ Apoptosis ↓ Mitochondrial function ↑	AML12 cells	Wang et al. (2025)
FA	Cadmium	Prevention	Cadmium concentration, AST, ALT, ALP, LDH ↓ TPC, SOD, CAT, TTH, TSH, GSH, TAC, GPx ↑ LOOH, NO, PCC, MDA, TOS, OSI ↓ HSP70, Cox-2, TNF-α ↓	Body weight ↑ Food and water consumption ↑ Liver damage score ↓ Inflammation and necrosis ↓ Antioxidant capacity ↑	Rat	Sanjeev et al. (2019)
FA	Iron	Prevention	Non-heme iron level, MDA ↓ GSH, SOD, Nrf2, GPX4, FPN, HO-1, NQO1↑ FtH, STAT3, SOCS3, FoxO1 ↓	Antioxidant capacity ↑ Oxidative damage ↓ Inflammation ↓	Mouse	Kose et al. (2023)
FAS	Iron	Prevention	Non-heme iron level, MDA, FtH ↓ GSH, SOD ↑ Nrf2, GPX4, FPN, HO-1, GCLC, NQO1↑	Antioxidant capacity ↑ Oxidative damage ↓ Inflammation ↓	Mouse	Kose et al. (2023)
FA and FAS	Iron	Prevention	ROS, MDA ↓ GSH, SOD ↑	Antioxidant capacity ↑ Oxidative damage ↓ Cell viability ↑	HepG2 cells	Kose et al. (2023)
FA	Iron	Prevention	Iron content, AST, ALT, TC, TBA, Fasn, Acc, CPT1A, CTYP7A1 ↓ Hamp, Fpn, FXR ↑ Dmt1, Tfrc, Slc39a14 ↓	Body weight ↑ Liver index ↓ Lipid and bile acid metabolism disorders ↓	Mouse	Liang et al. (2024)
FA	Iron	Prevention	Iron content, MDA, TC, TG, ↓ PPARα, Acox1, Adipoq, Bsep, Shp ↑ Fasn, Acc, Cyp7a1 ↓	Lipid and bile acid metabolism disorders ↓ Cell viability ↑	AML12 cells	Liang et al. (2024)
SF	Iron	Prevention	Liver index, ALT, AST, MDA, GSSG, ROS ↓ SOD, GSH-Px, CAT, GSH, ratio of GSH/GSSG, GSH + GSSG, MMP ↑	Apoptosis ↓ Mitochondrial swelling ↓	Rat	Qiao et al. (2016)
FA	Fluoride	Prevention	AST, ALT, ALP, ACP, GGT, LDH, CK, Bilirubin, Cholesterol, TG, LDL, VLDL ↓ TBARS, LOOH, NO↓ HDL, TP, ALB, Globulin, SOD, CAT, GPx, GSH, Vit.C↑	Body weight gain ↑ Histopathological changes↓ Lipid metabolism disorder↓ Antioxidant capacity ↑	Rat	Panneerselvam et al. (2013)
FA	NaF	Prevention	Liver index, TBARS, CD, LDH ↓ TG, Cholesterol, LDL, AST, ALT, ALP ↓ SOD, CAT, HDL↑ The binding energy of the SOD-FA interaction was better than the SOD-H_2_O_2_ interaction, while the CAT-FA interaction was better than the CAT- H_2_O_2_ interaction	Histopathological changes↓ Oxidative damage ↓	Rat	Das et al. (2023)
FA	Avermectin	Prevention	ALT, AST, TC, TG, LDL, MDA↓ HDL, IL-10, TGF-β1, Bcl-2↑ IL-1β, IL-6, TNF-α, Bax, Caspase3, Caspase9 ↓ SOD, T-AOC, CAT, GSH, GSH-Px ↑	Histopathological score ↓ Oxidative stress ↓ Inflammation ↓ Apoptosis ↓	Carp (*Cyprinus carpio*)	Sun et al. (2024)
FA	Difenoconazole	Prevention	ALT, AST, TC, TG, LDL ↓ HDL, IκBα, IL-10, TGF-β1, Bcl-2↑ Nrf2, nqo1, ROS, MDA ↓ NF-κB, IL-1β, IL-6, TNF-α ↓ Bax, Caspase3, Caspase9, P53, Cyt-c ↓ SOD, T-AOC, CAT, GSH, GSH-Px ↑	Oxidative stress ↓ Inflammation ↓ Mitochondrial Apoptosis ↓	Carp (*Cyprinus carpio*)	Hu Q. et al. (2024)
FA	Diosbulbin B	Prevention	ALT, AST, DRPAs formation ↓	Liver damage ↓	Mouse	Chen et al. (2023)
FA	Diosbulbin B	—	Metabolic rate of DIOB ↓	DRPAs formation ↓	Rat liver microsome	Chen et al. (2023)
FA	Diosbulbin B	Prevention	ALT, AST, ALP, MDA, TNF-α, IFN-γ, MPO ↓ IκB ↑	Hepatocyte degeneration ↓ Lymphocyte infiltration ↓ Apoptosis ↓	Mouse	Niu et al. (2016)
FA	Diosbulbin B	Therapeutic administration	ALT, AST, MDA, LPO↓ CuZn-SOD, CAT ↑	Liver vacuolar degeneration↓ Subcutaneously tumor weight↓	Mouse subcutaneously inoculated with mouse sarcoma S180 tumor cells	Wang et al. (2014)
Nano sesame protein isolate encapsulated FA	Acrylamide	Prevention	ALT, AST, ALP, GGT, TB ↓ MDA, NO, IL-6, TNF-α, Caspase3 ↓ TP, ALB, GSH, GPx, CAT, SOD ↑ Tail length, DNA fragmentation ↓	Body weight gain ↑ Feed efficiency ratio ↑ Liver index ↓ Liver morphological changes↓ DNA damage ↓ Apoptosis ↓	Rat	Essa et al. (2025)
FA	Cyclosporine	Prevention	ALT, AST, ALP, TB ↓ MDA, Nitrite, Protein carbonyl ↓ Nrf2, HO-1, CAT, SOD, GPx, GSH, Bcl-2 ↑ NF-κB, TNF-α, IL-1β, Bax, Caspase3 ↓	Oxidative stress ↓ Inflammation ↓ Apoptosis ↓ Histopathological score ↓	Rat	Nouri et al. (2023)
SF	Prednisolone	Prevention	GST, GPT, MDA ↓	Lipid peroxidation ↑ Membrane fluidity of microsome and mitochondria↓	Mouse	Wu et al. (1995)
FA	Cisplatin	Prevention	ALT, AST, NO, MDA, COX, IL-1β, cleaved caspase3↓ CAT, SOD, NF-κB↑	Histopathological changes ↓	Rat	Esmat et al. (2022)
FA	Gamma-radiation	Prevention	GOT, GPT, ROS, MDA ↓ p-JAK1, p-STAT3, Ferrous ions ↓ GSH, GPx4, SLC7A11, Nrf2↑	Histopathological score ↓ Iron deposition ↓ Oxidative damage ↓ Inflammation and fibrosis ↓	Rat	Gawish et al. (2024)
FA	Gamma-radiation and/or Aluminium chloride	Prevention	ALT, AST, ALP, GGT, TB, DB, TC, TG, LDL, VLDL, MDA, PCC, Fe, Zn ↓ HDL, CAT, GPx, SOD, GSH, Cu ↑	Hepatic function ↑ Dyslipidemia ↓ Histological alterations ↓	Rat	Salem et al. (2016)

##### 2.1.1.2 Ferulic acid alleviate acetaminophen-induced liver injury

Acetaminophen (APAP) is known as a widely used antipyretics and analgesics. When taken overdose, APAP can cause acute liver injury. As shown in Table 1, a research has approved (*in vivo and in vitro*) that FA could effectively ameliorate APAP-induced acute liver injury in mice via reducing oxidative damage, balancing MMP, up-regulating adenosine monophosphate-activated protein kinase (AMPK) phosphorylation, promoting protective autophagy, and inhibiting apoptosis (Wu et al., 2022). Another *in vivo* study with mice found that FA alleviated APAP-induced liver apoptosis and inflammation through up-regulating antioxidants (SOD, CAT, GSH) and inhibiting Caspase3, CYP2E1, TNF-α, IL-1β, TLR4, p-IRAK1, p-IκB, and p-P38 (Yuan et al., 2016). As a safe and active salt of ferulic acid, sodium ferulate (SF) has activity of scavenging free radical. Early study has proved that mice pretreated with SF were resistant to acetaminophen-induced liver damage. SF prevented acetaminophen-induced mice liver injury by inhibiting the activity of ALT, reducing the depletion of liver glycogen and glutathion, increasing the activity of GST, inhibiting the production of MDA, and diminishing the membrane fluidity of liver microsome and the mitochondria (Wang and Peng, 1994).

##### 2.1.1.3 Ferulic acid alleviates mycotoxin-induced liver injury and

**FIGURE 2:**
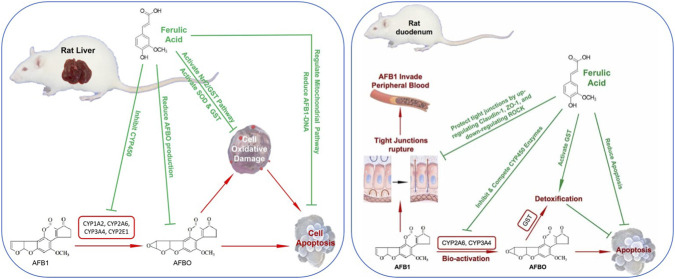
The mechanism of ferulic acid in mitigating aflatoxin B1-induced liver damage (left panel reproduced from Wang X. et al., 2021, licensed under CC-BY-NC-ND; right panel reproduced from Wang X. et al., 2022, licensed under CC-BY-NC-ND).

The effect of FA on the liver of male Wistar rats exposed to aflatoxin B1 (AFB1) has been studied by our team. AFB1 is a pre-carcinogen. AFB1 is metabolized by cytochrome P450 (CYP450) enzyme to produce AFB1-8,9-epoxide (AFBO), which is a highly toxic metabolite with cytotoxicity and carcinogenicity. As shown in Figure 2 and Table 1, FA treatment reduced AFB1-induced liver morphological changes, suppressed the increased serum index (ALT, AST, GGT, ALP, TBA) caused by AFB1, and reduced the generation of AFBO. The AFB1-induced high expression of CYP450 enzymes (CYP1A2, CYP2A6, CYP3A4, and CYP2E1) was significantly reduced by FA. The results of molecular docking between FA and CYP2A6 showed that they are highly adaptable and interactive. The amino acids of GLN360, ASP364, PRO431, and ALA445 in CYP2A6 protein structure can be bound by both AFB1 and FA. The equilibrium dissociation constant between AFB1 and CYP2A6 is similar as the equilibrium dissociation constant between FA and CYP2A6, which indicated that FA and AFB1 compete each other when binding to CYP2A6. FA significantly inhibited the production of ROS and MDA, activated Nrf2/GST pathway and antioxidant enzymes (SOD and GST) in rats livers poisoned by AFB1. Nrf2 is a nuclear transcription factor that plays an important role in antioxidant production by promoting antioxidant genes expression such as glutathione S transferase (GST) and superoxide dismutase (SOD) (Abed et al., 2015). FA restored the high expression of pro-apoptotic genes (p53, bax, cyt-c, caspase-9, caspase-3) and the low expression of anti-apoptotic gene (bcl-2) induced by AFB1 exposure. FA significantly reduced the account of apoptotic liver cells in rats fed with AFB1. This study proved that FA has an obvious inhibitory effect on AFB1-induced liver injury in rats by inhibiting and competing CYP450 enzymes, promoting the antioxidant pathway Nrf2/GST, and regulating the mitochondrial apoptosis pathway (Wang X. et al., 2021). It has been reported that FA can prevent AFB1 from invading the peripheral blood and extraintestinal organs via protecting the duodenal barrier, so as to reduce the liver toxicity (Wang X. et al., 2021). FA protected the duodenal barrier by reducing duodenal epithelial cells apoptosis, protecting tight junctions (TJs) between duodenal epithelial cells via increasing the tight junction proteins expression (Claudin-1 and ZO-1) and inhibiting ROCK protein expression, reducing the bio-activation of AFB1 through inhibiting and competing CYP450 enzymes (CYP2A6 and CYP3A4) in duodenal epithelial cells, and accelerating the detoxification of AFB1 via enhancing the activity of GST.

Compared to the white rice, brown rice has high content of FA. FA extracted from brown rice has good performance in protecting mouse liver against the toxicity of AFB1 (Wang et al., 2025). *In vivo* (with mice) and *in vitro* (with AML12 cells) experiments proved that FA mitigated AFB1-induced hepatic injury via a potential mechanism associated with antioxidant capacity enhancement, mitochondrial function restoration, and attenuation of apoptosis (Table 1). A recent study explored the potential of FA against mycotoxin-induced oxidative stress in Caco-2 cells (Rădulescu et al., 2025). The cells were exposed to AFB1 and ochratoxin A (OTA), and administrated with FA. The results showed that FA could mitigate oxidative stress by regulating lipid peroxidation and protein oxidation, decreasing 4-hydroxy-2-nonenal (4-HNE), increasing the activity of SOD, and preserving thiol groups by scavenging ROS. The decrease of polyubiquitinated Nrf2 and the improvement of SOD activity indicated that FA stabilized Nrf2 by delaying its degradation and enhancing its antioxidant role. These findings indicate that FA can counteract mycotoxin-induced hepatic and intestinal oxidative damage, highlighting the need for further investigation into FA’s long-term effects (Table 1).

#### 2.1.2 Ferulic acid and its derivatives protect liver from environmental toxins (cadmium, iron, fluoride, avermectin, and difenoconazole)

##### 2.1.2.1 Ferulic acid mitigates liver damage induced by cadmium

Cadmium (10 mg/kg) was given subcutaneously for 15 and 30 days to induce hepatic toxicity in rats. The cadmium concentration in liver was significantly increased. The addition of FA (50 mg/kg) significantly reduced the concentration of cadmium in liver. Cadmium-poisoned rats showed reduced body and organ weights, increased food and water consumption, and elevated rectal temperatures. In addition, cadmium reduced the total protein content (TPC) and the level of antioxidants {SOD, CAT, total thiols (TTH), total free sulfhydryl groups (TSH), GSH, total antioxidant concentration (TAC), GPx}, increased the activity of hepatotoxic marker enzymes (AST, ALT, ALP, LDH), the levels of biomarkers associated with oxidative damage {lipid hydroperoxides (LOOH), NO, protein carbonyl content (PCC), MDA, total oxidant status (TOS), oxidative stress index (OSI)}, and inflammation markers {heat shock protein 70 (HSP70), COX-2, TNF-α}. Whereas these parameters changes were obviously alleviated in FA-treated rats (Sanjeev et al., 2019). This study gives a clue that FA can protect rat liver from cadmium toxicity by inhibiting cadmium from entering the liver and reduce the production of free radical.

##### 2.1.2.2 Ferulic acid and its derivative protect liver from iron induced damage

**FIGURE 3:**
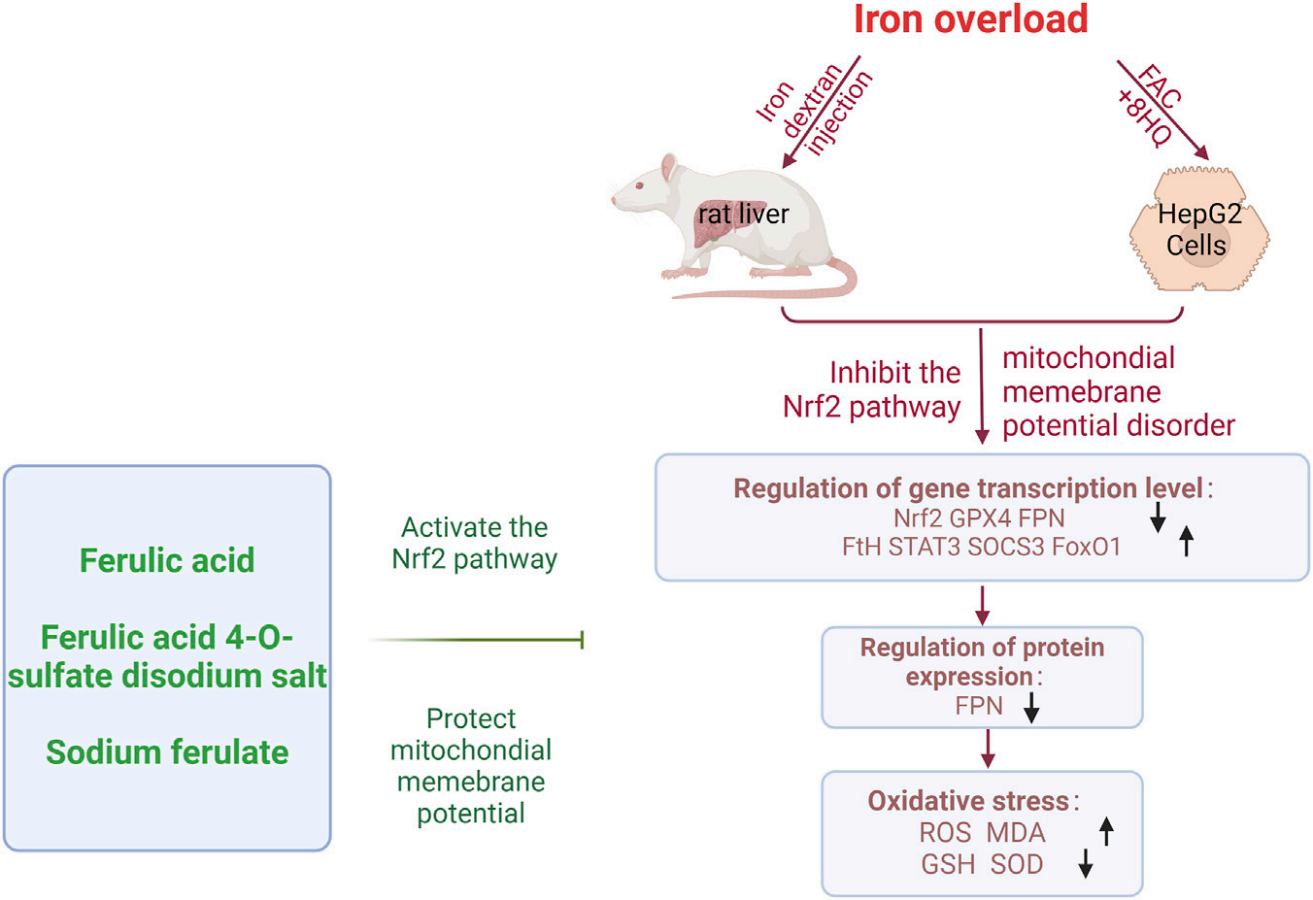
Protective effects of ferulic acid and its derivatives on liver injury induced by iron overload.

Liver is an iron storage organ. Therefore, liver is susceptible to oxidative stress induced by excess iron. As antioxidants, FA and its metabolite ferulic acid 4-O-sulfate disodium salt (FAS) were used to protect hepatocytes under iron overload conditions in mouse and HepG2 cells (Kose et al., 2023). As revealed in Figure 3, the results revealed that FA and FAS reduced the content of ROS and MDA, and increased the level of GSH and SOD in HepG2 cells administrated with FAC + 8-hydroxyquinoline (8HQ), which can cause rapid iron overload damage. In mice injected with iron dextran, non-heme iron level in liver was significantly increased. FA and FAS treatment obviously elevated the level of endogenous antioxidants (GSH and SOD), and decreased the level of MDA in mice livers under iron overload condition. Iron overload induced low transcription level of Nrf2, glutathione peroxidase 4 (GPX4), ferritin transporter protein (FPN), and high transcription level of ferritin heavy chain (FtH), signal transducer and activator of transcription 3 (STAT3), suppressor of cytokine signaling 3 (SOCS3), forkhead box O1 (FoxO1) in mice liver were significantly reversed by FA and FAS. The protein expressions of Nrf2, GPX4, heme oxygenase 1 (HO-1), glutamate-cysteine ligase catalytic subunit (GCLC), and NADPH quinone oxidoreductase 1 (NQO1) in iron overloaded mice livers were not statistically changed compared to the control group. But FA and FAS treatment statistically promoted the protein expressions of Nrf2, GPX4, HO-1, GCLC, and NQO1. In the same trend as the transcription level, FPN protein expression was obviously reduced in iron overloaded mice compared to the control group, but was significantly increased in FA and FAS treated mice. This study proved from both *in vivo* and *in vitro* that FA and FAS can protect liver from iron overload induced damage via activating Nrf2 pathway. In addition, as shown in Table 1, another *in vivo* study proved that FA can target fatty acid synthase (FASN) and CYP7A1 to inhibit lipid and bile acid metabolism disorders and further protect mice liver from iron overload induced injury (Liang et al., 2024).

SF has been used for cell protection. Iron-overloaded mice showed increased liver-to-body weight ratio, increased transaminase level, obvious hepatocellular apoptosis, high level of ROS, mitochondrial swelling, and mitochondrial membrane potential (MMP) disorder. Long-term (15 weeks) of SF supplementation obviously attenuated the impaired liver function caused by iron-overload. Iron-overload induced high liver-to-body weight ratio, and elevation in transaminase and cell apoptosis were significantly reduced by SF treatment. The mechanism of SF alleviating iron-overload caused liver injury is to inhibit oxidative stress by elevating the activities of antioxidant enzymes, decreasing ROS, protecting the structure of mitochondria, and balancing MMP (Qiao et al., 2016). The protective effects of FA and its derivatives on liver injury induced by iron overload were summarized in Table 1.

##### 2.1.2.3 Ferulic acid reduces fluoride-induced liver damage

*In vivo* study, *in vitro* study, and *in silico* analysis have predicted and proved the protective effect of FA on fluoride-induced hepatotoxicity and its possible mechanisms. Fluorine (25 mg/L) orally induced hepatotoxicity in rats for 12 weeks. Liver injury was assessed using serum markers AST, ALT, ALP, acid phosphatase (ACP), glutamyltransferase (GGT), lactate dehydrogenase, bilirubin, lipid profile, TPC levels, and histopathological tests. From the reduction of lipid hydroperoxides and NO level, the recovery of enzymatic and non-enzymatic antioxidant levels, and the restore of TPC in rat livers treated with FA in comparison with rats exposed to fluorine, it was concluded that FA treatment significantly reduced the degree of histological lesions and lipid peroxidation induced by fluorine (Panneerselvam et al., 2013). Female albino rats treated with 200 ppm/kg sodium fluoride (NaF) showed high levels of thiobarbituric acid-reactive substances (TBARS) and conjugated diene (CD), and low levels of CAT and SOD in liver. The biomarkers of liver injury in serum, such as triglyceride, cholesterol, low density lipoprotein (LDL), AST, ALT, ALP, and lactate dehydrogenase (LDH), all increased. Histopathological test revealed that NaF caused dilation of central vein and sinusoidal, erythrocyte infiltration, and inflammatory cells infiltration in liver. These results indicate that NaF causes liver injury through promoting lipid peroxidation, and inhibiting the production of antioxidants in liver (Das et al., 2023). FA (10 mg/kg and 20 mg/kg body weight) co-administered with NaF remarkably protected rat liver. The antioxidative status was promoted and the lipid peroxidation level was decreased in liver, which resulted in the histomorphological changes of liver were obviously alleviated. Based on the above scientific phenomena, molecular docking was used to simulate the interactions between FA and antioxidant enzymes. In-silico analysis showed that FA protects liver from NaF-induced liver oxidative damage via conjugating with the active side of CAT and SOD, which can prevent the binding of NaF to these antioxidant enzymes. Furthermore, the *in silico* analysis was proved by *in vitro* study. The *in vitro* study showed that CAT and SOD existed in the liver tissue homolysate could be activated by FA to breakdown H_2_O_2_. But NaF could inhibit the activity of CAT and SOD. These studies give evidence of FA alleviating fluoride-induced liver injury via regulating the activity of antioxidant enzymes and inhibiting lipid peroxidation.

##### 2.1.2.4 The detoxic effect of ferulic acid on avermectin and difenoconazole

The pesticide residues will pollute the water environment and poison the health of fish. The most recent studies with carp (*Cyprinus carpio*) have investigated the toxicity effect of two pesticides avermectin (AVM) and difenoconazole (DFZ), and the detoxic effect of FA on AVM and DFZ. AVM is a veterinary drug used to treat animal parasitic diseases. Carp livers exposed to AVM for 30 days showed oxidative damage, inflammation, and cell apoptosis. Dietary addition of FA obviously alleviated AVM-induced carp liver injury by ameliorating oxidative damage, inhibiting inflammation, and reducing hepatocellular apoptosis (Hu Q. et al., 2024). DFZ is a triazole fungicide used to protect crops such as tomatoes, soybeans, bananas, cereal crops, etc. DFZ residue presents in the environment poses a significant risk to non-target organisms. The experimental data showed that carp exposed to DFZ revealed significant liver damage. In carp livers exposed to DFZ, the levels of ROS and lipid peroxidation product MDA were increased, while the total antioxidant capacity (T-AOC), the activity of CAT, and the content of GSH were decreased; The transcriptional levels of nuclear factor NF-κB and pro-inflammatory cytokines (IL-1β, IL-6, TNF-α) were elevated, while the transcription levels of anti-inflammatory cytokines (IL-10 and TGF-β1) were reduced; The protein relative expressions of Bax and cytochrome C (Cyt c) and the transcription levels of p53, bax, caspase9, caspase3 were up-regulated, while the protein relative expression and the mRNA relative expression of Bcl-2 were down-regulated. Adding FA to the feed of carp exposed to DFZ, the production of ROS and MDA, the expression of pro-inflammatory cytokines (IL-1β, IL-6, TNF-α), and the expression of pro-apoptotic genes (p53, bax, caspase9, caspase3, cyt c) involved in mitochondrial pathway were inhibited. Moreover, the antioxidant capacity of liver, the expression of anti-inflammatory cytokines (IL-10 and TGF-β1), and the expression of anti-apoptotic gene Bcl-2 were promoted. These results gave a clue that FA can protect carp from DFZ-induced liver injury via improving the antioxidant capacity of liver, inhibiting inflammation process, and inhibiting mitochondrial pathway apoptosis (Sun et al., 2024).

#### 2.1.3 Ferulic acid and its derivative protect liver from chemicals and radiation (diosbulbin B, acrylamide, cyclosporine, prednisolone, and radiation)

##### 2.1.3.1 Preventive effect of ferulic acid on diosbulbin B-induced liver injury

It was discovered that FA has protective effect on Diosbulbin B (DIOB)-induced acute liver injury. Mice were given FA (20 mg/kg, 40 mg/kg, 80 mg/kg) orally once daily for 6 days. Diosbulbin B (250 mg/kg) was given orally on the last day. Elevated levels of alanine aminotransferase (ALT), aspartate aminotransferase (AST), and alkaline phosphatase (ALP) in serum are considered as bio-markers of liver injury. FA (40 mg/kg, 80 mg/kg) reduced Diosbulbin B-induced elevation of ALT, AST, and ALP in mice serum. Histological test result showed that FA (80 mg/kg) alleviated hepatocyte degeneration and lymphocyte infiltration caused by diosbulbin B. Moreover, FA (80 mg/kg) decreased the amount of apoptotic cells in liver. FA (40, 80 mg/kg) reduced the increased hepatic MDA content, the high levels of serum TNF-α and interferon-γ (IFN-γ), and the activity of myeloperoxidase (MPO) in liver which caused by Diosbulbin B. FA (80 mg/kg) reversed the decrease in kappa B inhibitor (IκB) expression induced by Diosbulbin B. These results proved that FA can prevent acute liver injury induced by Diosbulbin B via inhibiting intrahepatic oxidative damage, inflammation, and hepatocyte apoptosis (Niu et al., 2016). Another research reported that FA can reduce Diosbulbin B-induced intense lipid peroxide (LPO), and increase the expression and the activity of copper- and zinc-containing superoxide dismutase (CuZn-SOD) and catalase (CAT) in mice livers. Liver vacuolar degeneration caused by Diosbulbin B was obviously reversed by FA administration. These results indicated that FA can alleviate liver oxidative injury caused by Diosbulbin B via inhibiting LPO and activating the antioxidant enzymes. Although Diosbulbin B can induce liver damage, but it has function of inhibiting tumor growth. Interestingly, FA was shown to enhance Diosbulbin B-induced tumor growth inhibition in mice. Experimental mice were subcutaneously inoculated with mouse sarcoma S180 tumor cells to establish tumor models. FA (8 mg/kg), diosbulbin B (32 mg/kg), or diosbulbin B (32 mg/kg) + FA (8 mg/kg) by intragastric administration for 12 days starting from 24 h after tumor inoculation. Compared to the mice inoculated with tumor cells but without FA or diosbulbin B treatment, FA, diosbulbin B, and FA + diosbulbin B treatment all statistically reduced the sizes of subcutaneously tumors. The anti-tumor effect of FA + diosbulbin B is better than FA alone and diosbulbin B alone treatment. This phenomenon gives an evidence that the combination of FA and Diosbulbin B can not only synergize anti-tumor growth, but also alleviate the hepatotoxicity of Diosbulbin B to normal liver (Wang et al., 2014). Therefore, when diosbulbin B is used for anti-tumor, FA can be combined to reduce the hepatotoxicity of diosbulbin B and enhance the anti-tumor effect. A most recent review reported that, as a traditional Chinese medicine, D. bulbifera contains compounds included primarily terpenoids, steroids, and phenolics. On one hand, D. bulbifera has a broad spectrum of pharmacological activities (antitumor, antioxidant, antimicrobial, antidiabetic, and immunomodulatory effects) depends on its high content of FA. On the other hand, D. bulbifera causes hepatotoxicity through its high concentration of Diosbulbin B. Its mechanism involves CYP450 activation, generating oxidative stress, mitochondrial dysfunction, and hepatobiliary transporters inhibition. Strategies such as herbal compatibility and processing, co-administration with protective agents (especially FA), show preclinical promise in reducing toxicity while preserving efficacy. This review gives a clue that Diosbulbin B-containing herbs are highly safe in combination with FA-containing herbs. Which means potential neutralizing effect of FA on the toxicity of Diosbulbin B (Zhang et al., 2026). A recent research found that FA can prevent DIOB-induced liver damage via inhibiting covalent modifications on proteins (Chen et al., 2023). Diosbulbin B can be metabolized by CYP450 enzymes to generate reactive metabolites (DIOB-RMs), which can covalently bind to proteins and result in hepatoxicity. It was found from *in vivo* experiment that the content of DIOB RM-protein adducts (DRPAs) positively correlate to the severity of hepatotoxicity. FA inhibited the production of DRPAs and suppressed DIOB-induced high ALT and AST levels in serum. *In vitro* experiment proved that FA was able to reduce the metabolic rate of DIOB. Thus, It was concluded that FA could ameliorate DIOB-induced liver damage via reducing the production of DRPAs.

##### 2.1.3.2 Ferulic acid alleviates acrylamide-induced liver toxicity

A recent study explored the potential of nano-encapsulated FA in sesame protein isolate (SPI) as a therapy for acrylamide (ACR) induced liver injury in rats (Essa et al., 2025). Rats treated with 20 mg/kg/day ACR for 2 weeks showed decreased body weight gain, total food intake, feed efficiency ratio, serum γ-GT activity, serum TP level, serum ALB level, and antioxidants (GSH, GPx, CAT, SOD). Rats treated with ACR also showed increased relative liver weight, liver enzymes (ALT, AST, ALP) level in serum, bilirubin level in serum, oxidative damage indicators (MDA, NO), inflammatory factor (IL-6, TNF-α), and DNA damage. These changes indicated that ACR can induce rat liver damage through improving oxidative damage, inhibiting the production of antioxidants, inducing inflammation, and causing DNA damage. Compared with the rats fed 20 mg/kg/day ACR, rats fed with 20 mg/kg/day ACR and 0.1% FA and 5% sesame protein in nano-encapsulated form showed increased body weight gain, total food intake, feed efficiency ratio, serum γ-GT activity, serum TP level, serum ALB level, antioxidants (GSH, GPx, CAT, SOD), and decreased relative liver weight, liver enzymes (ALT, AST, ALP) level in serum, bilirubin level in serum, oxidative damage indexes (MDA, NO), inflammatory factor (IL-6, TNF-α), DNA damage. But the levels of total food intake, GSH, GPx, CAT, SOD, MDA, NO, ALT, AST, bilirubin, IL-6, TNF-α, and DNA damage did not recovered to normal. This study proved that nano-encapsulated FA in SPI can effectively mitigate the liver toxicity of ACR via regulating oxidative stress, inhibiting inflammation, and modulating DNA damage.

##### 2.1.3.3 Protective effect of ferulic acid and its derivative on liver damage induced by cyclosporine, prednisolone, and cisplatin

Long-term administration of cyclosporine exerts devastating effect on liver cells. A recent study reported that FA treatment not only can protect rat liver from cyclosporine-induced oxidative damage through activating Nrf2/HO-1 signaling axis; But also can inhibit cyclosporine-induced liver inflammation by reducing the expressions of pro-inflammatory cytokines such as NF-κB, TNF-α, and IL-1β; Reduce cyclosporine-induced hepatocytes death via shifting the equilibrium between the expression levels of pro-apoptotic proteins to anti-apoptotic proteins (Nouri et al., 2023). Early study has proved that mice pretreated with SF were resistant to liver damage induced by prednisolone. SF partially prevented prednisolone-induced mice liver damage via reducing the content of MDA, and stabling the membrane fluidity of liver microsome (Wu et al., 1995). Cisplatin (CIS) is a classic platinum chemotherapy drug with broad-spectrum anti-tumor. Its hepatotoxicity limits its clinical application. A study investigated the protective role of FA against CIS-prompted hepatotoxicity in rats (Esmat et al., 2022). The results showed that, CIS administration caused increased oxidative stress, aminotransferase activities, COX-2 and IL-1β transcription, caspase-3 enzyme activity, and decreased anti-oxidative responses, NF-κB-p65 and caspase-1 transcription. The administration of FA ameliorated CIS-induced hepatotoxic effect. Its mechanism is attributed to the possession of anti-oxidative, anti-inflammatory, and anti-apoptotic capabilities (Table 1).

##### 2.1.3.4 Ferulic acid protects against γ-radiation caused liver damage

The latest report shows that FA can effectively alleviate γ-radiation caused liver damage in rats (Gawish et al., 2024). As shown in Table 1, Rats received 6Gy γ-radiation showed obvious histopathological changes in liver and hepatic function disorder. FA administration effectively inhibited γ-radiation caused liver damage and restored the tissue structure and function of liver. FA inhibited γ-radiation induced hepatic inflammatory response and prevented liver fibrosis through blocking JAK/STAT signaling pathway. FA alleviated γ-radiation induced liver oxidative damage via up-regulating the Nrf2 signaling pathway, promoting the expression of several antioxidants, reducing lipid peroxidation and ROS production. Moreover, FA inhibited γ-radiation induced hepatocellular ferroptosis by improving the expression of GPX4 and SLC7A11, and down-regulating iron aggregation. A previous study reported that oral administration of FA ameliorated γ-radiation-induced hepatic function impairment, dyslipidemia and hepatic histological alterations; reduced MDA and PCC levels; elevated the activiy of CAT, GPx, and SOD as well as GSH level; increased liver Cu concentration; and decreased the concentrations of Fe and Zn in rat liver (Salem et al., 2016).

### 2.2 Ferulic acid and its derivatives can prevent and treat alcoholic liver disease

Alcoholic liver disease (ALD) is caused by acute or chronic ethanol intake. ALD can induce abnormal lipid retention in the liver, increase fatty acid synthesis, and ultimately lead to hepatic steatosis (Stickel et al., 2017; Lu and Wang, 2025). FA was reported to have hepatoprotective effect on ethanol-induced liver damage in a dose dependent manner (10, 20, 40 mg/kg body weight) in rats (Rukkumani et al., 2004). The most recent research reported that FA extracted from Angelica sinensis (Oliv.) Diels can ameliorate lipid metabolism disorder in ALD (Lu and Wang, 2025). C57BL/6 mice and HepG2 cells were used to investigate the effect of FA extracted from Angelica sinensis (Oliv.) Diels on ALD. The results showed that FA could protect mice from ethanol-induced liver damage through ameliorating fat deposition and inhibiting oxidative damage. It was demonstrated that FA could ameliorate ethanol-induced lipid metabolism imbalance via promoting the activation of AMPK, Acetyl-CoA carboxylase (ACC), PI3K, AKT, and further regulating their down-stream proteins such as SREBP1, FASN, CPT1, and PPAR. In other words, FA could alleviate ethanol-induced liver injury and lipid metabolism disorder via AMPK/ACC and PI3K/AKT pathways.

MFA is also known as caffeic acid dimethyl ether. Its chemical name is 3,4-dimethoxy-ben-zene acrylate. *In vivo* and *in vitro* studies have investigated the role of MFA in the treatment of ALD. The results of *in vivo* study showed that MFA treatment reduced serum ALT and AST activity, increased the levels of SOD, CAT, GSH, GSH-Px, T-AOC, and reduced the concentration of MDA and ROS. Histopathological examination of the liver revealed that MFA reduced alcohol-induced cytoplasmic vacuolization, necrosis, and inflammatory cell infiltration in mice. MFA can reduce alcohol-induced high levels of TG, TC, LDL, ethanol dehydrogenase (ADL), and aldehyde dehydrogenase (ALDH). MFA inhibited alcohol-induced high expression of the inflammatory factors TNF-α, monocyte chemotaxis protein 1 (MCP-1), IL-1β, and IL-6. MFA inhibited the mRNA and protein expressions of NOX4 and CYP2E1, reduced the ratio of Bax/Bcl-2. MFA inhibited the activation of caspase-3 and caspase-9, downregulated the levels of p-JNK, p-p38 MAPK, and p-ERK in the liver. In conclusion, MFA has protective effect on ALD, which may be related to its capability of antioxidant, anti-inflammatory, and regulation of NOX4/ROS-MAPK signaling pathway (Li et al., 2017). Another research have successfully developed a rat model of alcoholic liver disease through chronic alcohol feeding. The rats were fed a diet containing 5% ethanol (w/v) for 16 weeks. The ALT and AST levels were increased in the ALD model rats, along with the typical histopathological changes such as steatosis, necrosis, apoptosis, inflammation, etc. MFA treatment not only reduced the serum transaminases levels, but also improved the liver histopathological changes (fat deposition and inflammatory response). In addition, MFA reduced TC and TG levels in serum and liver, decreased LDL-C level, and increased HDL-C level. These results indicating that MFA alleviated liver steatosis in rats with ALD.

Ethanol administrated L-02 cells were used as an *in vitro* model to analyze the protective effect of MFA against hepatic steatosis. The findings showed that ethanol-induced increase of TC and TG in L-02 cells is in line with previous *in vivo* findings. TG and TC levels were decreased after MFA treatment, which indicating that MFA can inhibit ethanol-induced lipid accumulation. *In vitro* and *in vivo* studies both found that MFA attenuated ethanol-induced hepatocellular damage through miR-378b-mediated activation of phosphatidylinositol 3-kinase (PI3K)/AKT pathway (Cheng et al., 2018; Cheng et al., 2019; Zhang Y. et al., 2022). Moreover, ethanol intake was found to induce lipid deposition in ALD rats through the inhibition of AMPK and FoxO1 signaling pathways (Roy et al., 2010; Shearn et al., 2013; Mandal et al., 2014; Xing et al., 2018). Ethanol inhibited lipid oxidation in rats by down-regulating the expression of SIRT-1, PPAR-α, and CPT-1α, which leads to lipid deposition. After MFA treatment, the above adverse effects of ethanol were reversed and liver steatosis in rats was alleviated. In conclusion, MFA has a clear protective effect against ethanol-induced hepatic steatosis.

**FIGURE 4:**
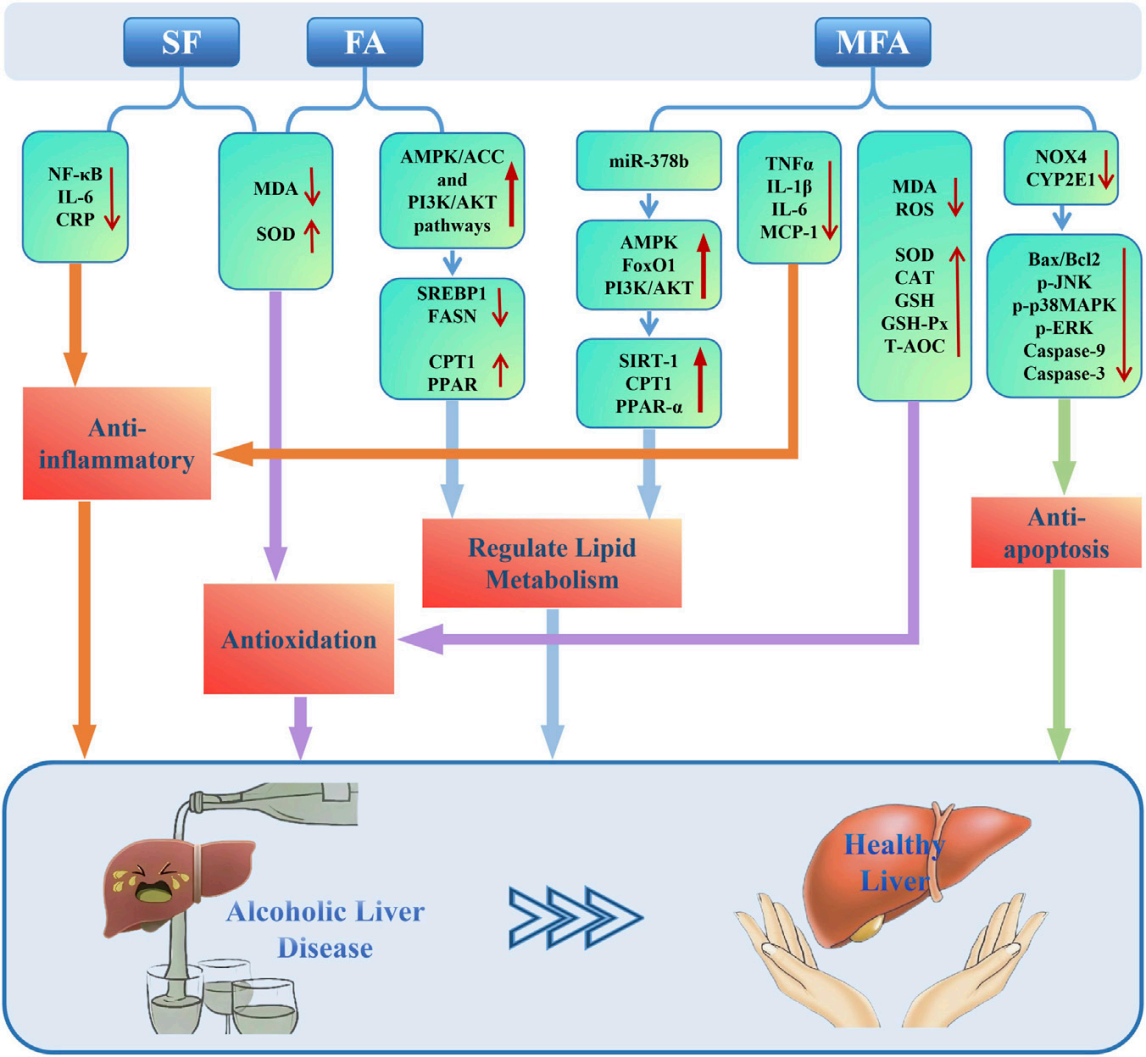
The effect of ferulic acid and its derivatives on alcoholic liver disease.

It was reported that SF can protect liver from ethanol-induced liver damage via promoting the activity of SOD, reducing the production of MDA, inhibiting the expression of C-reactive protein (CRP), IL-6, NF-κB, and then resulted in the reduction of ALT, AST, TG, and the recovery of liver histopathological changes (Pei et al., 2014). The combination of SF and oxymatrine (OMT) showed better hepatoprotective effect than SF alone in the liver of mice exposed to ethanol. This research indicated that SF and OMT combination can promote the hepatoprotective effect synergistically and that antioxidation and anti-inflammatory effects might be involved in the protective mechanism. The effect of FA, MFA and SF on alcoholic liver disease was summarized in Figure 4.

### 2.3 Prevention and treatment effect of ferulic acid on non-alcoholic fatty liver disease

Non-alcoholic fatty liver disease (NAFLD) is characterized by the excessive accumulation of fat in liver cells due to the imbalance between energy output and input. The major pathological changes caused by NAFLD include obesity, type 2 diabetes, and dyslipidemia (Petta et al., 2009). There are a wide range of NAFLD patients. Natural phenolic acid and its derivatives are considered as a promising candidate for the prevention and treatment of NAFLD, and FA is an active phenolic acid (Wang et al., 2020; Akinyelu et al., 2025; Cui et al., 2026).

The classical *in vitro* model of hepatic steatosis is oleic acid (OA) treated HepG2 cells. The accumulation of excess OA is associated with hepatic steatosis and can reflect the status of intracellular lipid deposition. A recent study showed that the lipid accumulation of the hepatocytes after 50 g/mL FA treatment was decreased by 23.1% compared with the OA treated cells. In HepG2 cells, the inhibitory effect of FA on OA-induced lipid accumulation was comparable to that of 10 μM γ-glutethimide, which is an antioxidant drug already used in liver protection. Hepatic steatosis and hepatitis have been found to be associated with increased expression of ERK and JNK, both of which belong to the MAPK family and play an important role in regulating hepatocyte energy homeostasis. The *in vitro* data supported that the beneficial effect of FA on fatty liver is due to the down-regulation of ERK 1/2 and JNK 1/2/3 expressions (Wang Z. et al., 2021). High-mobility family protein B1 (HMGB1) is a pro-inflammatory factor released by hepatocytes after free fatty acid treatment, and can serve as a biomarker of NAFLD. FA was found to inhibit the expression of HMGB1 in OA-stimulated HepG2 cells. This phenomenon demonstrated the therapeutic effect of FA on NAFLD.

*In vivo* studies proved that sufficient intake of FA (0.5%) could treat obesity and obesity-related diseases. FA controlled the weight and improved glucose homeostasis, lipid profiling, and hepatic steatosis of C57BL/6 mice fed with HFD through maintaining the self-renewal capacity of embryo stem cells (ESCs) and adipose-derived mesenchymal stem cells (ADMSCs) (Wang et al., 2019; Cho and Park, 2020). Mice fed a HFD orally for 12 weeks showed more excessive energy intake than mice fed with control diet. Inbred mouse strain C57BL/6 has a host-genetic predisposition and showed body weight gain after feeding on a HFD. Frequent exposure to high-energy foods also leads to liver steatosis, rapid weight gain, and abnormal circulating lipids. FA (100 mg/kg/day) administration significantly attenuated HFD induced hepatic steatosis and lipid accumulation. HFD-induced high level of hepatic TG was decreased by 33.5% in FA-treated mice. The results of metabolic cage studies showed that the energy expenditure during dark phases was increased by 11.5%. As the target of PPAR-α and the rate-limiting enzymes of fatty acid oxidation and ketone body biosynthesis, CPT1A, ACOX1 and HMGCS2 protein expressions were elevated by FA. It was concluded that FA could prevent HFD-induced NAFLD in mice via activating PPAR-α pathway to promote energy expenditure and inhibit triacylglycerol accumulation in liver (Luo et al., 2022). In a study with rats, high-cholesterol and high-fat diet (HCHF) induced non-alcoholic steatohepatitis (NASH) can be attenuated by FA via ROCK/NF-κB signaling pathway (Wei et al., 2021). HCHF-induced oxidative damage in liver was alleviated by FA through inhibiting the formation of ROS and MDA, and improving the activity of SOD. In addition, FA administration restored HCHF-caused liver inflammation via down-regulating the activity of ROCK/NF-κB signaling pathway, and then downregulated the downstream target molecules IL-1β, IL-6, and TNF-α. Another study found that treatment with FA for 17 weeks failed to reverse HFD-induced liver damage in C57BL/6 mice. Long-term intake of HFD has been shown to increase obesity and severe liver injury characterized by steatosis and inflammation. Adipocyte size and steatosis with inflammation did not alleviate after treatment with FA (Wang Z. et al., 2021). FA enhanced lipid metabolism by up-regulating the phosphorylation of AMPKα and down-regulating the expression of SREBP1 and ACC1 (Gu et al., 2021). The beneficial effect of FA on metabolic changes in mice and rats fed a HFD was independently reported by different scholars, and the dose of FA varied in different studies. The dose and mode of administration of FA may lead to different observations (Liu et al., 2024).

It has been suggested that multiple injuries act together to induce NAFLD, which is a more accurate explanation of the pathogenesis of NAFLD than the previous one of a single factor (Brunt et al., 2015; Cui et al., 2026). In NAFLD induced by the combination of multiple factors, the intestinal flora plays an important role through the gut-liver axis (Ma et al., 2019; Fu et al., 2023). The intestinal microbiota is regulated by nutritional overload and obesity. Nutritional overload and obesity promote metabolic disturbances that allow bacterial products and whole bacteria to be transferred to the peripheral circulation through increased intestinal permeability caused by reduced tight junction proteins expression. These interrelated and parallel events trigger inflammation and provoke immune cell infiltration in different areas, particularly the liver and adipose tissue. FA significantly altered HFD-induced relative abundance of intestinal flora, including Firmicutes, Bacteroidetes and Proteobacteria. Firmicutes and *Bacteroides* were the most abundant flora in the mouse intestine (Porras et al., 2017). A high proportion of Firmicutes and a low proportion of *Bacteroides* are beneficial to polysaccharide catabolism and fat deposition. The higher the ratio of Firmicutes/*Bacteroides*, the greater the negative impact on the intestine health (Bäckhed et al., 2004). It was found that FA can reduce the ratio of Firmicutes/Bacteroidetes and increase the proportion of Bacteroidetes. Porphyromonas aeruginosa was found to break down tryptophan to produce I3A (tryptophan metabolite). Thus, the increase in the Proteobacteria was consistent with plasma I3A levels. The aromatic hydrocarbon receptor (AHR) recognizes foreign organisms and natural compounds such as tryptophan metabolites. I3A in the intestine can bind to the AHR in the liver via the intestine-hepatic axis. I3A regulates the expression of fatty acid synthase (FASN) and cholesterol regulatory element binding protein (SREBP-1c) through the activation of AHR. FASN and SREBP-1c are key proteins that regulate lipid and fatty acid synthesis, respectively. It was reported that HFD can cause dysbiosis of the intestinal flora, reduce I3A concentration in plasma, and reduce AHR activity in the liver. These changes increased the expression of FASN and SREBP-1c in the liver, which accelerating the deposition of lipid and cholesterol in the liver and ultimately the formation of NAFLD. The use of FA can reverse these changes (Ma et al., 2019; Cui et al., 2026). Studies in Arbor Acres broilers also proved the mitigation effect of FA on fat deposition in liver through regulating lipid metabolism related genes (CYP7A1, SIRT1, AMPK, PGC-1α, PPAR, CPT-1α, FAS, and SREBP-1c), and gut microbiota (Zhang et al., 2025; Long et al., 2026).

**FIGURE 5:**
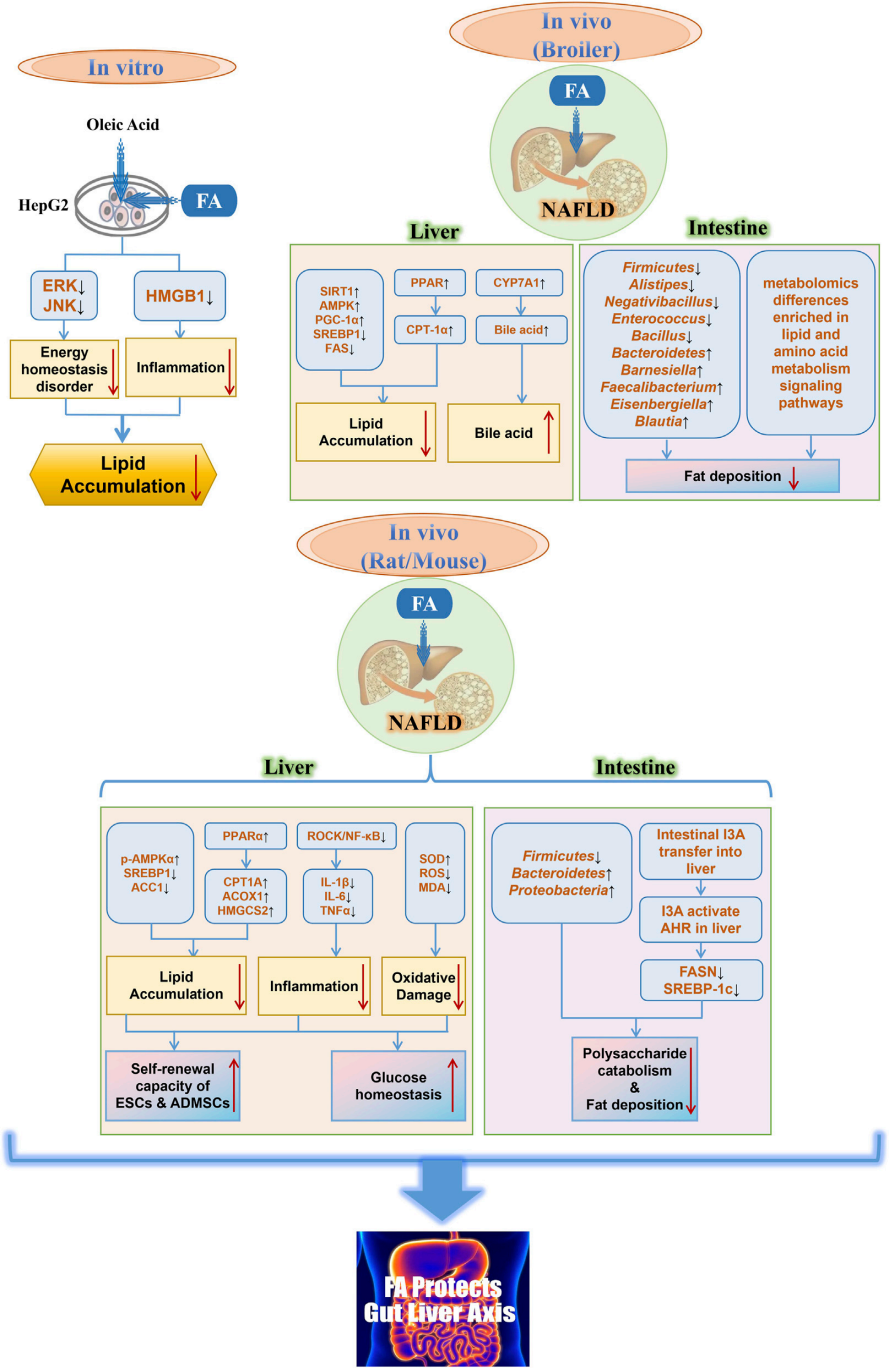
Effect of ferulic acid on non-alcoholic fatty liver disease.

Multiple factors contribute to the progression of NAFLD to NASH, including insulin resistance, liver lipid accumulation, sedentary lifestyles, high fat diets, obesity, and intestinal flora disorder (Lucas et al., 2018). These factors act in tandem with intestinal or adipose tissue to promote liver inflammation. In conclusion, the treatment of FA for NAFLD may be closely related to the lipid and fatty acid metabolism disorder induced by remodeling of the intestinal microbiota. However, further and deeper research is needed. The effect of FA on NAFLD was summarized in Figure 5.

### 2.4 Ferulic acid and its derivative protect against liver fibrosis and liver cancer

Chronic inflammation induced by different pathogenic factors leads to liver fibrosis, which subsequently progresses into malignant liver diseases with high mortality, such as cirrhosis and hepatocellular carcinoma (HCC). There are a series of pathological alterations occur in the liver when the balance between synthesis and degradation of the extracellular matrix (ECM) is disrupted. According to the current research findings, the typical characteristics of hepatic fibrosis are the activation of hepatic stellate cells (HSC) and ECM disorder. The molecular mechanisms included inflammatory factors, growth factors, chemotactic factors, and molecules related to oxidative stress (Kisseleva and Brenner, 2021). In a recent study, hepatic fibrosis was induced in mice by administrating with Con A for 4 weeks. At the same time, treated with FA at different doses (5, 10, and 20 mg/kg/day). The data demonstrated that FA had a palliative effect against liver fibrosis in a dose-dependent manner. This was manifested by the recovery of liver markers and the improvement of liver tissue structure along with the regression of fibrosis in the FA-treated mice. FA could inhibit oxidative damage, promote the activities of antioxidant enzymes through the Nrf2/HO-1 signaling pathway, suppress NF-κB, IL-1β, and TNF-α levels. In addition, FA restored Con A-induced increase of TGF-β, Smad3, α-SMA, and hydroxyproline content. These results gave an evidence that FA could attenuate Con A-induced liver fibrosis via activating antioxidant signaling pathway Nrf2/HO-1, inhibiting inflammatory signaling pathway NF-κB/IL-1β, and suppressing fibroblast proliferation signaling pathway TGF-β/Smad3 (Elazab and Hsu, 2024). A previous study also proved this (Mu et al., 2018). Iron oxide (Fe_3_O_4_) nanoparticles (IONPs) and ferulic acid (FA) coencapsulated poly (lactic-co-glycolic acid) (PLGA) nanoparticles (NPs), followed by surface modification with cRGD peptides (cRGD-PLGA/IOFA) enhanced the hepatoprotective function of FA in liver fibrosis via ancting on macrophages and HSCs (Hu Z. et al., 2024). Another study demonstrated that FA effectively protected mice from CCl_4_ induced liver fibrosis (Wu et al., 2021). As a critical energy sensor, AMPK is regulated by the phosphorylation of liver kinase LKB1 and the dephosphorylation of protein tyrosine phosphatases (PTPs). After 4 weeks of CCl_4_ administration, mice were treated with different doses of FA (25, 50, and 100 mg/kg) for another 4 weeks. In FA treated groups, activity of ALT and AST in serum were inhibited, the expression of molecules involved in fibrogenesis was reduced. These phenomena indicated that CCl_4_-induced liver inflammation and liver fibrosis were ameliorated. Additionally, the anti-inflammatory and anti-fibrotic effects of FA were abrogated by a specific AMPK inhibitor without the involvement of LKB1. Intermolecular interaction tests proved that FA could directly bind to PTP1B and inhibit its activity, which resulted in AMPK phosphorylation. FA inhibited CCl_4_-induced hepatic oxidative stress, macrophages infiltration, and the activation of hepatic stellate cells through AMPK phosphorylation. Mechanistically speaking, the beneficial effects of FA on liver fibrosis induced by CCl_4_ were due to its activation of PTP1B-AMPK signaling pathways. In recent years, a research team invented a new derivative FA11, which can inhibit TGF-β1-induced LX-2 proliferation and lead to cycle arrest and apoptosis, block HSCs activation, suppress epithelial-mesenchymal transition (EMT), and alleviate collagen deposition (Xue et al., 2023). FA11 reduced macrophage infiltration and prevented macrophage polarization to a profibrotic phenotype. FA11 reversed the phosphorylation of TGF-β1 signalling, as well as FGFR1 signalling.

HCC is a common primary liver cancer with a 5-year survival rate of only 18%. FA has been reported to have antitumor effect on lung cancer, breast cancer, liver cancer, and intestinal cancer (Bao et al., 2023). A recent research has explored the antitumor effect and mechanism of FA in rats with HCC caused by thioacetamide administration (Abdulal et al., 2024). Rats with HCC were given 50 mg/kg FA three times per week for 16 weeks. The results showed that FA treatment increased the survival rate of rats with HCC. Alpha-fetoprotein (AFP) is highly expressed in liver cancer and many tumors, and it is mainly used as a biomarker of primary liver cancer in clinic. The level of AFP in FA-treated rats serum was reduced, which means liver function was improved and liver cancer was controlled. The sizes of hepatic nodules were suppressed. Morphological examination results revealed that FA encouraged the appearance of vacuolated cytoplasm, reduced necrotic nodules and apoptotic nuclei in HCC. The transcription and expression of anti-hypoxia induced factor-1α (HIF-1α), NF-κB, TNF-α, mTOR, STAT3, cMyc, and cyclin D1 were decreased by FA. These results indicated that FA can inhibit HCC-induced hypoxia via inhibiting the expression of HIF-1α, suppress tumor growth via down-regulating the expressions of mTOR, STAT3, cMyc, and cyclin D1, inhibit inflammation via down-regulating NF-κB and TNF-α. Carbonic anhydrase IX (CAIX) is a transmembrane protease which can be elevated and activated in the tumor cell membrane by hypoxia. The activated CAIX involved in cancer cell invasion and metastasis. A study reported that an FA-triazole compound had good properties in inhibiting the activity of liver tumor cells in a dose-dependent manner via inhibiting CAIX. This study indicated that FA can be used as a CAIX inhibitor for a targeted therapy of liver cancer (Aneja et al., 2020). In addition, FA treatment before radiotherapy can enhancing the radiotherapy sensitivity in liver cancer cells by increase the level of ROS and regulating the antioxidant status of cancer cells (Das et al., 2019). A research gave evidence that FA could inhibit the proliferation, and increase the apoptosis and autophagy of liver tumor cells via promoting the protein expressions of Caspase-3, cleaved-Caspase-3, Beclin-1, LC3-II/LC3-I, PINK-1, and Parkin (Wang J. et al., 2022).

Solid tumor growth is promoted by growth-stimulatory polypeptide growth factors and their receptors, as well as by hormones secreted by chronic stress-regulated neuroendocrine systems (Abubakar et al., 2024; Sharma, 2024; Bazzal et al., 2025). Solid tumor growth is dependent on angiogenesis induced by tumor cells. Studies have shown that blocking tumor angiogenesis suppresses tumor growth, invasion, and metastasis. In recent years, the research on tumor treatment through inhibiting tumor angiogenesis has received wide attention. As the derivative of FA, SF has the effect of antitumor blood vessel growth. Researchers have found that SF acts to inhibit the growth and angiogenesis of H_22_ liver tumors in mice. H_22_ tumor cells and vascular endothelial cells were treated with various doses of SF (≤160 mg/L). SF does not have a direct cytotoxic effect on H_22_ tumor cells, and can not directly inhibit the growth of endothelial cells (Xu et al., 2007). Orthotopic liver cancer model was established by injecting H_22_ tumor cells into mice liver. The results of histopathological test and immunohistochemistry experiment showed that different doses of SF treatment can effectively reduce the microvascular density in tumor tissues, and significantly inhibit the transcription and expression of vascular endothelial growth factor (VEGF) in liver tumor. VEGF has been reported as the most important pro-angiogenic factor. These results suggest that SF inhibits liver tumor growth and angiogenesis in mice mainly by inhibiting the mRNA transcription of VEGF, and causing a decrease in VEGF production (Chen et al., 2008). Most recent research synthesized a new trans-FA (TFA) derivative as a potential anticancer agents and VEGFR-2 inhibitor (Mohie et al., 2026). TFA double-O-alkylated with benzyl bromide, then was dicarbonylated, then added with OCH_3_ to produce a derivative of TFA named TFA-4e. TFA-4e demonstrated exceptional cytotoxicity against HCC cells (HepG2, Huh7, and Hep3B). In WI-38 fibroblasts, TFA-4e showed 23-fold greater potency than TFA and 2-fold superiority to doxorubicin when maintaining minimal toxicity. Mechanistic studies showed that TFA-4e downregulated anti-apoptotic molecules {α-fetoprotein (AFP), BCL-2, MCL-1, and γ-carboxyprothrombin}, upregulated pro-apoptotic caspase-3 and the tumor suppressor P53. TFA-4e exhibited inhibitory activity against VEGFR-2 as good as Sorafenib. These results suggest that TFA-4e can block the development of HCC via promoting cell apoptosis and reduce angiogenesis in liver tumor.

**FIGURE 6:**
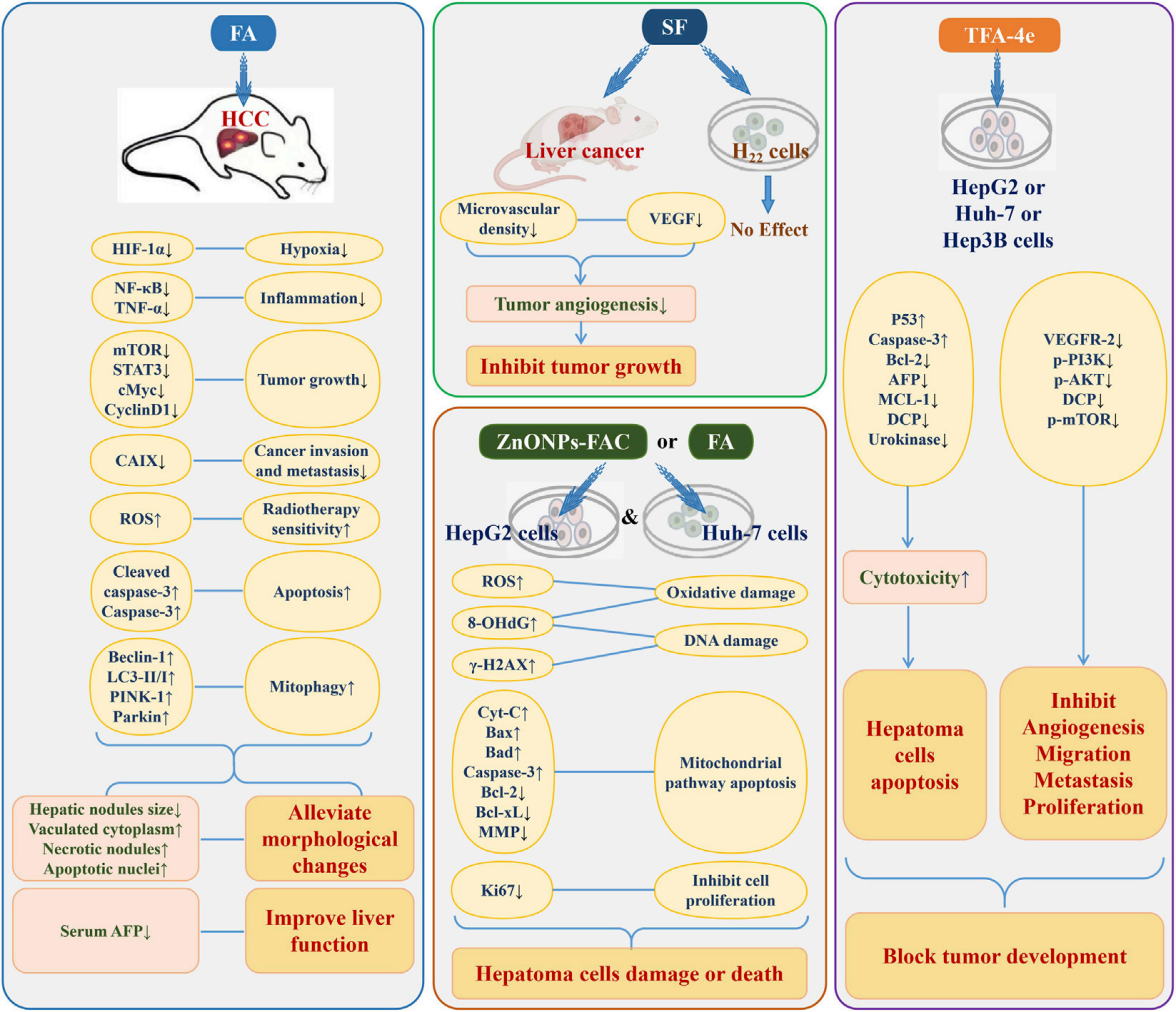
Effect of ferulic acid and its derivative on liver cancer. Note: FA means ferulic acid; SF means sodium ferulate; ZnONPs-FAC means ZnO nanoparticle-ferulic acid; TFA-4e: trans-FA double-O-alkylated with benzyl bromide, then was dicarbonylated, then added with OCH3 to produce a derivative of TFA named TFA-4e.

A nanomedicine research using human hepatocellular cancer cells (Huh-7 and HepG2) and rats found the therapeutic potential of ZnO nanoparticle-ferulic acid (ZnONPs-FAC) conjugate on HCC (Ezhuthupurakkal et al., 2018). As a promising method, chemotherapeutics based on ZnO nanoparticles (ZnONPs) has been emanated and well explored. It can maximize the therapeutic synergy and help facilitating the discovery of new multitargeted combinations. ZnONPs-FAC was characterized with computational, spectroscopic, and microscopic techniques. In the experiments with Huh-7 and HepG2 cells, the anticancer potential of ZnONPs-FAC was evaluated via assessing cell viability, morphological test, the content of ROS, MMP, comet assay, cell cycle test, immunofluorescent staining of cell proliferation marker (Ki67), DNA oxidative damage marker (8-OHdG), and the key DNA damage regulatory factor (γ-H2AX). The results showed that the content of ROS, the expressions of 8-OHdG, γ-H2AX, Cyt-C, Bax, Bad, Caspase-3 in Huh-7 and HepG2 cells were increased, the expressions of Ki67, Bcl-2, and Bcl-xL were down-regulated, and the MMP change was recovered by ZnONPs-FAC, ZnONPs, and FA treatment. These results indicated that ZnONPs-FAC, ZnONPs, and FA can inhibit the proliferation of cancer cells, and induce liver cancer cells death through oxidative damage and mitochondrial pathway apoptosis. The damage effect of ZnONPs-FAC on liver cancer cells was better than ZnONPs and FA, which means ZnONPs-FAC conjugate has synergistic therapeutic effect on HCC *in vitro*. In DEN-induced HCC animal model, ZnONPs-FAC treatment effectively alleviated the histopathological changes and reduced the level of tumor marker protein. *In vivo* research gave a clue that ZnONPs-FAC treatment can suppress HCC. Effect of ferulic acid and its derivative on liver cancer was summarized in Figure 6.

### 2.5 Ferulic acid in combination with other drugs protect liver health

The hepatoprotective effects of ferulic acid combined with drugs are summarized in Table 2.

**TABLE 2:** Ferulic acid in combination with other drugs protect liver health.

Combination drug or cell	Liver disease	Modeling method	Signaling pathway	Apparent change	Animal or cell	References
Astragalin IV + FA	Liver fibrosis	Bile duct ligation	Nrf2 in nucleus ↑ Antioxidant enzymes ↑ GSK-3β ↓ ROS ↓ TGF-β1, TGF-βR, Smad3, Smad4 ↓	Collagen formation ↓ HSCs activation ↓	Rat	Zhao et al. (2020)
Gallic acid + FA	Liver fibrosis	Thioacetamide	SOD, CAT ↑ MDA ↓ TGF-β1, Smad3, p-Smad3 ↓ miR-21 ↓ miR-30, miR-200 ↓	ALT, AST, ALP ↓ Protect the integrity of liver tissue	Rat	Hussein et al. (2020)
Bone marrow mesenchymal stem cells + FA	Liver fibrosis	CCl4	miR-19b-3p ↑ α-SMA, COL1-A1 ↓ RhoA/ROCK pathway ↓	Inhibit cytoskeletal rearrangement of HSCs HSCs activation ↓ Alleviate liver fibrosis	Rat	Zhang R. et al. (2022)
Bone marrow mesenchymal stem cells + FA	Liver fibrosis	Hepatic stellate cell-T6	miR-19b-3p ↑ α-SMA, COL1-A1 ↓ RhoA/ROCK pathway ↓	Inhibit cytoskeletal rearrangement of HSCs HSCs activation ↓	Hepatic stellate cell-T6	Zhang R. et al. (2022)
Oxymatrine + SF	Ethanol-induced liver damage	Ethanol-induced liver damage	ALT, AST, TG, MDA, CRP, IL-6, NF-κB↓ SOD↑	Histopathological score ↓	Mouse	Pei et al. (2014)
Low doses of γ-irradiation + FA	Cisplatin-prompted hepatotoxicity	Cisplatin-prompted hepatotoxicity	ALT, AST, NO, MDA, COX, IL-1β, cleaved caspase3↓ CAT, SOD, NF-κB↑	Histopathological changes ↓	Rat	Esmat et al. (2022)
Ellagic + FA	Gamma radiation -induced liver damage	Gamma radiation	ALT, AST, ALP, GGT, TB, DB, TC, TG, LDL, VLDL, MDA, PCC, Fe, Zn ↓ HDL, CAT, GPx, SOD, GSH, Cu ↑	Hepatic function ↑ Dyslipidemia ↓ Histological alterations ↓	Rat	Salem et al. (2016)
Ellagic + FA	Aluminium chloride-induced liver damage	Aluminium chloride	ALT, AST, ALP, GGT, TB, DB, TC, TG, LDL, VLDL, MDA, PCC, Fe, Zn ↓ HDL, CAT, GPx, SOD, GSH, Cu ↑	Hepatic function ↑ Dyslipidemia ↓ Histological alterations ↓	Rat	Salem et al. (2016)
P-Coumaric Acid + FA	NAFLD	High-fat diet	ALT, AST, TG, Oil Red O, HDAC1, FABP, CD36 ↓ FA and p-CA bind to different sites of HDAC1	Body weight ↓ Morphological changes ↓ Lipid accumulation ↓	Mouse	Cui et al. (2022)
P-Coumaric Acid + FA	NAFLD	FFA	Oil Red O, Relative Bodipy intensity, TG, HDAC1, PPAR, FABP, CD36 ↓	Lipid accumulation ↓	HepG2 PLCPRF5 BEL-7402 cells	Cui et al. (2022)
Wogonin + FA	Cholestatic liver injury	α-naphthylisothiocyanate	ALT, AST, ALP, Sirius red positive area ↓ FD4 in serum, Goblet cells ↑ *Alistipes putredinis* ↓ *Clostridium spp.* ↑	Histopathological score ↓ Collagen accumulation ↓ Intestinal barrier ↑	Mouse	Luo et al. (2025)

#### 2.5.1 Astragalus agside cooperates with ferulic acid to alleviate liver fibrosis

Liver fibrosis, a response to chronic liver injury caused by any etiology, was initially thought to be irreversible and incurable. But it is currently known to be a dynamic process with significant regression potential. This finding provides new opportunities for the development of anti-fibrotic therapies. The activation of HSCs is a key event in the occurrence and development of liver fibrosis, and is also a major contributor to the collagen overdeposition regulated by a complex network of signaling pathways (Tsuchida and Friedman, 2017). Therefore, activated HSCs is an attractive target for anti-fibrotic therapy (Higashi et al., 2017). TGF-β is a powerful fibrotic factor. As the intracellular mediator of TGF-β, Smad protein plays a key role in HSCs activation, and stimulating the transcription of genes involved in collagen formation. TGF-β and Smad have potential to be therapeutic targets for liver fibrosis (Tang et al., 2017). Oxidative stress plays an important role in liver fibrosis formation. ROS can activate HSCs and promote fibrogenesis. By reviewing the historical role of several Chinese medicine formulas in the treatment of cirrhosis, it is found that Angelica blood tonifying soup (Astragalus, Angelica) and its active ingredients astragalin IV (AsIV) and FA may provide natural treatment for liver fibrosis (Xu et al., 2015). Thus, it is speculated that the combination of FA and AsIV have synergistic anti-fibrotic effects. In 2020, an *in vivo* study with bile duct-ligated cirrhotic rats gave evidence that AsIV synergized with FA to alleviate hepatic fibrosis (Zhao et al., 2020). Interestingly, the combination of AsIV and FA has better performance in inhibiting HSCs activation when compared with AsIV or FA alone. When AsIV synergized FA to protect against liver fibrosis, AsIV, but not FA, induced Nrf2 accumulation in the nucleus, negatively regulated glycogen synthase kinase-3β (GSK-3β) to synthesize antioxidant enzymes, scavenged ROS, and further inhibited the activation of HSCs. On the contrary, FA, but not AsIV, reduced the expressions of TGF-β1 and its receptor (TGF-βR), decreased the levels of Smad3 and Smad4, which led to the decrease of collagen formation and the suppression of HSCs activation. In conclusion, the molecular mechanism of AsIV and FA synergy in alleviating liver fibrosis induced by bile duct ligation is related to the activation of Nrf2 antioxidant system and the inhibition of the canonical TGF-β1 signaling pathway.

#### 2.5.2 Gallic acid and ferulic acid jointly inhibit liver fibrosis

The length of MicroRNAs (miR) is 19–24 nucleotides. They are non-coding RNA particles. They have important roles in cell differentiation, proliferation, immunity, and carcinogenesis. In the liver, some miRs have been identified as pro-fibrotic or anti-fibrotic molecules (Teng and Ghoshal, 2015; Noetel et al., 2012). Researchers have studied the possible protective effects of gallic acid (GaA) and FA on the experimentally thioacetamide (TAA) induced liver fibrosis in rats (Hussein et al., 2020). The experimental rats were executed after 6 weeks of continuous GaA + FA, GaA alone, and FA alone treatments respectively. Liver tissue was examined for liver function, biomarkers of oxidative stress, and histopathological changes. The changes of TGF-β1/Smad3 signaling pathway and the expressions of miR-21, miR-30, and miR-200 were evaluated. The results showed that GaA alone administration, FA alone administration, and the combination of GaA and FA could significantly reduce the activities of serum ALT, AST, and ALP in TAA-treated rats, and protect the integrity of liver tissue. GaA treatment, FA treatment, and GaA + FA treatment enhanced the activities of liver antioxidant enzymes (SOD and CAT). Meanwhile, the content of MDA in liver recovered to normal level. The protein expressions of TGF-β1, p-Smad3, and total Smad3 in liver were all significantly reduced by GaA, or FA, or GaA + FA treatment. The expression of miR-21 was down-regulated, the expression of miR-30 and miR-200 were up-regulated in response to GaA and FA. In addition, compared to GaA alone treatment and FA alone treatment, the protective effect of GaA and FA combination treatment against TAA-induced hepatic fibrosis was better. These results indicated that GaA and FA can synergistically alleviate TAA-induced hepatic fibrosis by inhibiting TGF-β1/Smad3 signaling pathway, and differentially regulating the expression levels of miR-21, miR-30, and miR-200.

#### 2.5.3 The combination of ferulic acid and bone marrow mesenchymal stem cells to attenuate liver fibrosis

It has been reported that bone marrow mesenchymal stem cells (BMSCs) can alleviate liver fibrosis, but its effect is insufficient. FA has been reported to have good performance in treating liver fibrosis. In recent years, researchers have investigated the combination effect of FA and BMSCs on liver fibrosis with *in vivo* and *in vitro* experiments (Zhang R. et al., 2022). Male SD rats were used to establish animal models of liver fibrosis with CCl_4_. Rat models of liver fibrosis were treated with BMSCs alone, or FA alone, or a combination of the two. The therapeutic effect of the combination was significantly better than BMSCs or FA alone. BMSCs isolated from SD rat bone marrow (SD-BMSCs) and hepatic stellate cell-T6 (HSC-T6) were used to verify the specific mechanism of BMSCs and FA combination in alleviating liver fibrosis. A SD-BMSCs/HSC-T6 co-culture system was created and FA was used to treat activated HSCs. The results showed that BMSCs and FA can inactivate HSCs. Cytochalasin D is an inhibitor of actin polymerization. Angiotensin II is a vasoconstrictor. Cytochalasin D and angiotensin II were used to investigate whether BMSCs and FA inactivate HSCs through cytoskeletal rearrangement. The results showed that cytoskeletal rearrangement of HSCs was inhibited in the combination group. *In vivo* and *in vitro* studies revealed that the expression of profibrotic markers (α-SMA and COL1-A1) was decreased in the FA and BMSCs combination administrating rats and cells. Angiotensin II treatment increased COL1-A1 and α-SMA expression, while cytochalasin D treatment decreased COL1-A1 and α-SMA expression in HSCs. MiR-19b-3p was high expressed in BMSCs. As a participant in the migration of the cytoskeleton, RhoA/ROCK pathway was inhibited in the combination treatment group. The expression of COL1-A1, α-SMA, and RhoA/ROCK pathway molecules was decreased in the activated HSCs treated with a miR-19b-3p. These results indicated that miR-19b-3p inactivated HSCs via inhibiting RhoA/ROCK signaling pathway. FA administration decreased the expression of profibrotic markers in BMSCs with or without miR-19b-3p. Which means FA alone also has good performance in inhibiting fibrosis. In summary, this study proved that the combination therapy had better effects than FA or BMSCs alone. And the combination treatment alleviated HSC activation and liver fibrosis via inhibiting cytoskeletal rearrangement, providing miR-19b-3p to inactivate HSCs, and suppressing RhoA/ROCK signaling pathway. The important effect of FA in the combination therapy was its better inhibitory effects on HSC activation.

#### 2.5.4 Protective role of ferulic acid and low-doses of γ-irradiation against cisplatin-prompted hepatotoxicity

A study investigated the protective role of FA and low-doses of γ-irradiation (LDR) against CIS-prompted hepatotoxicity (Esmat et al., 2022). The results showed that, CIS administration upregulated oxidative stress, aminotransferase activities, transcriptions of COX-2 and IL-1β, activity of caspase-3, and downregulated anti-oxidative responses, and the transcriptions of NF-κB-p65 and caspase-1. FA alone and FA + LDR treatment effectively alleviated CIS-induced hepatotoxicity via inhibiting oxidative damage, inflammation, and cell apoptosis. The therapeutic effect of FA + LDR is better than FA alone.

#### 2.5.5 Ellagic and ferulic acids alleviate γ-radiation and aluminium chloride-induced liver damage

Oral intake of FA alone, Ellagic (EA) alone, and FA + EA alleviated hepatic function impairment, dyslipidemia, and hepatic histological alterations induced by γ-radiation or aluminium chloride (AlCl_3_) or γ-radiation + AlCl_3_. Its mechanism is through reducing MDA and PCC levels, improving the activiy of CAT, GPx, and SOD as well as GSH level, increasing liver Cu concentration, and reducing the concentrations of Fe and Zn in rat liver (Salem et al., 2016).

#### 2.5.6 Ferulic acid and P-coumaric acid synergistically attenuate non-alcoholic fatty liver disease

A study from China found that FA and p-coumaric acid (p-CA) can synergistically attenuate NAFLD (Cui et al., 2022). Cell lines HepG2, PLCPRF5, and BEL-7402 treated with free fatty acid (FFA) that include oleic acid (OA) and palmitic acid (PA) to establish *in vitro* model of liver steatosis. FA + p-CA administration decreased oil red O in cells, relative bodipy intensity in cells, TG level, and the expression of HDAC1, PPAR, FABP, CD36. Meanwhile, FA + p-CA reduced ALT, AST, TG levels, oil red O content in liver, and the expression of HDAC1, FABP, CD36 in mice liver tissues. Molecular docking result gave an evidence that FA and p-CA could bind to different sites of HDAC1. Finally, FA + p-CA treatment suppressed final body weight and TG content, and alleviated liver dysfunction in NAFLD mice. This report found that PPAR, which is positively related to HDAC1, was inhibited by FA + p-CA, and further suppressed FABP and fatty acid translocase (CD36). It suggests that FA + p-CA alleviate NAFLD by inhibiting FFA uptake via the HDAC1/PPARG axis.

#### 2.5.7 Ferulic acid and wogonin synergetically protect agaist cholestatic liver injury

Cholestasis is a bile flow disorder correlates to many liver diseases. It has been reported that gut microbiota disturbances, especially increased Alistipes putredinis (A. putredinis) and decreased *Clostridium* spp (C. spp.), closely link to cholestatic liver injury. Wogonin (Wog) is a flavonoid extracted from *Scutellaria baicalensis Georgi*. It can suppress proinflammatory cytokine and inhibit oxidative stress. Wog also demonstrates anticancer and neuroprotective effect. A recent report found that FA + Wog effectively alleviated cholestatic liver injury in mice through reduce collagen accumulation and protect intestinal barrier (Luo et al., 2025). *Alistipes putredinis* administration exacerbated liver inflammation via breaking intestinal barrier integrity and facilitating bacterial translocation. FA + Wog treatment down-regulated the concentration of A. putredinis in mice intestine, and reversed the bad influence of A. putredinis. *Clostridium* spp. is a beneficial bacterium that can enhance bile salt hydrolase activity and bile acid excretion. FA + Wog treatment upregulated the concentration of *Clostridium* spp. in mice intestine. In addition, FA + Wog administration increased the count of goblet cells and elevated the level of FD4 in mice serum, which indicated that FA + Wog synergetically protected the intestinal barrier.

### 2.6 Ferulic acid exerts an anti-diabetic effect by affecting liver metabolism

It has been reported that treatment of diabetic animals with FA restored blood glucose level, serum insulin level, glucose tolerance, and insulin resistance to normal levels; Hepatic glycogen concentration, glycogen synthase, and glycokinase activity were significantly reduced; Glycogen phosphatase and the activities of glyconeogenase phosphoenpyruvate carboxykinase (PEPCK) and glucose-6-phosphatase (G6Pase) in liver were increased to normal levels. FA significantly reduced the interaction between FoxO1 and the gluconeogenic enzyme genes’ (PEPCK and G6Pase) promoters (Narasimhan et al., 2015; Li et al., 2022; Akinyelu et al., 2025). FoxO1 is directly phosphorylated by the protein kinase Akt, which can be activated by the insulin receptor. The phosphorylation of FoxO1 results in the inactivation of FoxO1 and nuclear repulsion (Nakae et al., 2001). Insulin resistance in high-fat-diet fed animals can inhibit the phosphorylation and inactivation of FoxO1, resulting in uncontrolled gluconeogenesis and hepatic glycogenesis. In diabetic animals, the mRNA expression level of glucose neovinase and the interaction between FoxO1 and the promoters of the gluconeogenase genes were increased. However, administration of FA in diabetic animals improved the insulin signaling cascade, thereby promoting the phosphorylation of FoxO1 and degradation. Researchers also found that FA reduced the interaction between FoxO1 and the promoter of the gluconeogenic enzyme genes (G6Pase and PEPCK), downregulated the lipogenic enzyme FAS-1, and upregulated the lipolytic enzyme CPT-1 by regulating a series of transcriptional factors including HNF4α, FOXO-1, SREBP-1c, and PPAR-γ. FA’s ability to mitigate diabetes is achieved by activating the insulin receptor/PI3K/AKT pathway (Li et al., 2022). Treatment of type II diabetic rats with FA improves insulin sensitivity and hepatic glycometabolism, and inhibites glyproduction and insulin signaling modulators to maintain euglycemic homeostasis (Narasimhan et al., 2015).

Streptozotocin (STZ) can induce diabetes in experimental animals. It has been found that FA can significantly alleviate the lipid peroxidation induced by STZ in rat liver (Suzuki et al., 2002; Akinyelu et al., 2025). Studies have found that alternate oral administration of FA in STZ-induced diabetic rats significantly reduced the oxidative damage to the liver caused by diabetes. Liver GSH level was significantly reduced in diabetic rats, indicating the importance of this non-enzymatic antioxidant. Reduced glutathione (GSH-Px) and its oxidation counterpart oxidized glatathione (GSSG) constitute the main redox buffer system. GSH can interact directly with ROS as a non-enzymatic antioxidant through the SH group, or it can participate in the detoxification reaction of ROS as a cofactor or coenzyme. Simultaneous FA supplementation in STZ-treated rats restored serum and liver GSH levels to normal endogenous levels, suggesting that the protective ability of FA may be partially attributed to its effect on GSH. After FA supplementation, the antioxidant enzymes’ (SOD, CAT) activity were enhanced, the content of MDA was decreased, which indicating that these oxidoreductases played an important role during FA preventing diabetes (Akinyelu et al., 2025). It is hypothesized from these studies that FA can alleviate lipid peroxidation *in vivo* by directly scavenging free radicals, or via enhancing the activities of antioxidant enzymes which can effectively remove free radicals (Thyagaraju and Muralidhara, 2008). The experimental rats received FA supplementation (50 mg/kg body weight) 1 week before STZ treatment (60 mg/kg body weight, intraperitoneal) and 1 week after STZ treatment (continued for 12 weeks) respectively. The results showed that 50 mg/kg FA supplementation did not influence the glucose level and lipid profile, but alleviated liver oxidative damage. This study indicated that although liver oxidative damage was attenuated by FA, but it was not sufficient to change biomarkers of diabetes (Hilary et al., 2023). When diabetic rats were treated with FA at 150 or 300 mg/kg body weight for 5 weeks, significant decrease of blood glucose level and obvious increase of serum insulin level were observed. In addition, FA treatment elevated the levels of GSH, HDL-c, SOD, and the activity of CAT, while suppressed the levels of MDA, cholesterol, triglyceride, LDL, NO, and the activities of ALT, AST, lipase, glycogen phosphorylase, glucose-6-phosphatase, and fructose-1,6-biphosphatase in the liver (Salau et al., 2023). These results indicated that high dose of FA treatment (150 or 300 mg/kg body weight) performed better than low dose of FA treatment (50 mg/kg body weight) in alleviating diabetes. Moreover, the antidiabetic potentials of FA involved affecting glycometabolism and lipid metabolism in liver. Some plant extracts rich in FA also have antioxidative, antidiabetic, and hypolipidemic properties in STZ-induced diabetes (Latifi et al., 2019; Alharbi et al., 2022).

## 3 Conclusions and outlook

Although *in vivo*, *in vitro*, and *in silico* studies showed that FA is a promising liver protective agent, because FA has been proved to have good performance in liver protection via regulating oxidative stress, alleviating inflammation, promoting immune response, influencing CYP450 enzymes, inhibiting fibrosis, protecting intestinal barrier and flora balance, improving insulin signaling cascade, balancing lipid metabolism and glycometabolism, and inhibiting the growth and angiogenesis of liver tumors. But the protective effect of FA on the liver has not been studied enough. Many researchers now use FA as a substrate for the development of effective drugs for treating liver diseases. Developing the derivatives of FA, the nanoparticles of FA, and the combination application of FA with other drugs for the prevention and treatment of liver damage become a research hotspot, which attracted more and more attention and research. It is still a long way to explore the application value, molecular mechanism, and development potential of FA and its derivatives in protecting liver health.

## 4 Methods

**FIGURE 7:**
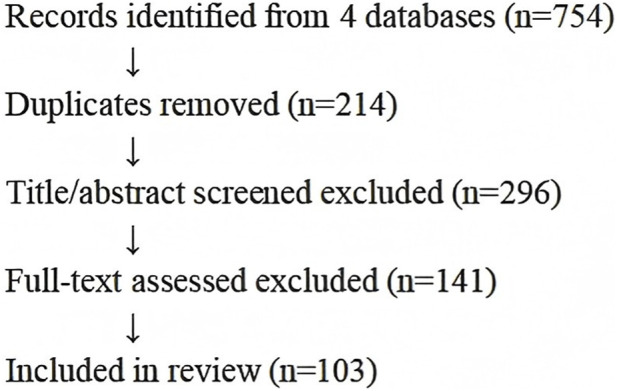
PRISMA flow diagram.

A systematic search was conducted in PubMed, Web of Science, Embase, and CNKI from January 2004 to March 2026. Search terms included: ferulic acid, hepatoprotection, liver injury, oxidative stress, inflammation, liver fibrosis, NAFLD, alcoholic liver disease, hepatocellular carcinoma, aflatoxin B1, cisplatin, Nrf2, TGF-β/Smad, JAK/STAT. Inclusion criteria were original *in vitro* or *in vivo* studies in English or Chinese investigating ferulic acid and its derivatives, and liver protection. Exclusion criteria were abstracts, duplicates, and non-hepatic studies. Two reviewers independently screened all articles. Risk of bias for animal studies was assessed using the SYRCLE tool. For *in vitro* studies, internal validity was assessed based on experimental design, controls, and reproducibility. A PRISMA flow diagram summarizes the study selection process. PRISMA flow diagram was shown in Figure 7.
